# African Programme for Onchocerciasis Control 1995–2015: Model-Estimated Health Impact and Cost

**DOI:** 10.1371/journal.pntd.0002032

**Published:** 2013-01-31

**Authors:** Luc E. Coffeng, Wilma A. Stolk, Honorat G. M. Zouré, J. Lennert Veerman, Koffi B. Agblewonu, Michele E. Murdoch, Mounkaila Noma, Grace Fobi, Jan Hendrik Richardus, Donald A. P. Bundy, Dik Habbema, Sake J. de Vlas, Uche V. Amazigo

**Affiliations:** 1 Department of Public Health, Erasmus MC, University Medical Center Rotterdam, Rotterdam, The Netherlands; 2 African Programme for Onchocerciasis Control, Ouagadougou, Burkina Faso; 3 School of Population Health, The University of Queensland, Brisbane, Australia; 4 Department of Dermatology, Watford General Hospital, Watford, United Kingdom; 5 Human Development Network, The World Bank, Washington, D.C., United States of America; 6 Independent Consultant, Enugu, Nigeria; Imperial College London, United Kingdom

## Abstract

**Background:**

Onchocerciasis causes a considerable disease burden in Africa, mainly through skin and eye disease. Since 1995, the African Programme for Onchocerciasis Control (APOC) has coordinated annual mass treatment with ivermectin in 16 countries. In this study, we estimate the health impact of APOC and the associated costs from a program perspective up to 2010 and provide expected trends up to 2015.

**Methods and Findings:**

With data on pre-control prevalence of infection and population coverage of mass treatment, we simulated trends in infection, blindness, visual impairment, and severe itch using the micro-simulation model ONCHOSIM, and estimated disability-adjusted life years (DALYs) lost due to onchocerciasis. We assessed financial costs for APOC, beneficiary governments, and non-governmental development organizations, excluding cost of donated drugs. We estimated that between 1995 and 2010, mass treatment with ivermectin averted 8.2 million DALYs due to onchocerciasis in APOC areas, at a nominal cost of about US$257 million. We expect that APOC will avert another 9.2 million DALYs between 2011 and 2015, at a nominal cost of US$221 million.

**Conclusions:**

Our simulations suggest that APOC has had a remarkable impact on population health in Africa between 1995 and 2010. This health impact is predicted to double during the subsequent five years of the program, through to 2015. APOC is a highly cost-effective public health program. Given the anticipated elimination of onchocerciasis from some APOC areas, we expect even more health gains and a more favorable cost-effectiveness of mass treatment with ivermectin in the near future.

## Author Summary

In 1995, the World Health Organization launched the African Programme for Onchocerciasis Control (APOC) with the aim to control morbidity due to the parasitic infectious disease onchocerciasis (river blindness). APOC aims to set up sustainable national control programs against onchocerciasis in 16 countries in sub-Saharan Africa, covering over 100 million people who are at risk for infection. The main control strategy is mass treatment with the drug ivermectin, which is donated by the pharmaceutical company Merck. Coordination of the mass treatment programs is made possible by financial contributions from donor and beneficiary countries. We estimated that between 1995 and 2010, APOC has had a huge impact on population health in sub-Saharan Africa, preventing 8.2 million years worth of healthy life from being lost due to disease and mortality, at a cost of about US$257 million. We predicted that this health impact will double during the subsequent five years, at a cost of about US$221 million. This makes APOC one of the most cost-efficient large-scale public health programs in the world. We may expect even greater health gains in the future, given the anticipated extension of the APOC mandate with the aim to eliminate infection where possible.

## Introduction

Onchocerciasis is caused by *Onchocerca volvulus*, a filarial nematode restricted to human hosts. The adult female worms reside in subcutaneous nodules where they produce millions of microfilariae during their on-average ten-year life span [1]. The microfilariae are found predominantly migrating through the skin and eyes and are transmitted by biting flies of the genus *Simulium* (the vector), an obligatory part of the parasite's life cycle. Onchocerciasis is responsible for a considerable burden of disease, mainly because of visual impairment, blindness, disfiguring skin lesions, and severe itching, which are the results of continuous exposure to microfilariae. Most of the global burden of onchocerciasis (>99%) is found in sub-Saharan Africa. In the West African savanna, where onchocerciasis is of a severely blinding form (savanna type), fear of blindness previously led to abandonment of fertile river basins. However, by now, onchocerciasis has been largely eliminated from West Africa by the Onchocerciasis Control Programme (1974–2002), which relied on intense vector control and mass treatment with the drug ivermectin [2].

In the more central and eastern parts of Africa, where onchocerciasis is usually of the less blinding form (forest type), there was no control or control only at a limited scale until the inception of the African Programme for Onchocerciasis Control (APOC) in 1995. APOC is a morbidity control program scheduled to be active until 2015, requiring that by that year, participating countries support and coordinate control measures independently. Since 1995, APOC has mapped infection with *O. volvulus* in 20 countries [3] and has coordinated interventions in 16 countries where onchocerciasis is considered a public health problem (Angola, Burundi, Cameroon, Central African Republic, Chad, Congo, Democratic Republic of Congo, Equatorial Guinea, Ethiopia, Liberia, Malawi, Nigeria, South Sudan, Sudan, Tanzania, and Uganda), covering endemic areas inhabited by about 71.5 million people in 1995. APOC's main strategy is to implement annual mass treatment with ivermectin.

Ivermectin kills microfilariae and permanently reduces the production of microfilariae by adult female worms, slowing down transmission and preventing morbidity [4], [5]. Annual mass treatment with ivermectin is implemented through a community-directed treatment approach, empowering communities to take responsibility for ivermectin delivery and to decide how, when, and by whom ivermectin treatment is administered. Mass treatment with ivermectin is enabled by donation of the drug by the pharmaceutical company Merck through the Mectizan Donation Program. Furthermore, coordination of the program is funded by donor countries (through the World Bank) and national onchocerciasis task forces (including beneficiary governments and non-governmental development organizations). To demonstrate APOC's importance, validate the efforts of endemic communities and national task forces, and maintain commitment of all stakeholders, it is essential to establish the health impact and cost of APOC.

Here, we present the estimated impact of APOC on population health and the costs involved up to 2010, with extrapolated trends up to 2015. An impact assessment would ideally be based on observed trends of infection and morbidity, but such longitudinal data are of limited availability in APOC areas. We therefore estimated trends of infection and morbidity based on APOC data of pre-control levels of infection and history of mass treatment, and literature-derived associations between infection and morbidity and the effect of treatment on infection and morbidity. For our calculations, we used ONCHOSIM, an established micro-simulation model for transmission and control of onchocerciasis [6], [7].

## Methods

### Project-population by endemicity category and project-specific history of control

The impact of APOC was estimated at project level (a project being an implementation unit for mass treatment with ivermectin), while taking account of the prevailing type of onchocerciasis (i.e., savanna versus forest or mixed forest/savanna, with different patterns of morbidity) and the project-specific history of control. Project populations were further stratified by endemicity groups, to take account of differences in the pre-control prevalence of morbidity (which is non-linearly associated with infection) and the potential impact of mass treatment (e.g., the impact is relatively lower in highly endemic areas due to more residual transmission between treatment rounds). We considered four endemicity levels: non-endemic (prevalence of onchocercal nodules in adult males <1%), hypoendemic (nodule prevalence ≥1% and <20%), mesoendemic (nodule prevalence ≥20% and <40%), and hyperendemic (nodule prevalence ≥40%).

We estimated the size of the population at risk for infection in the 107 geographical project areas covered by APOC, for the years 1995–2010 (see File S1). These estimates were based on records kept by community-appointed drug distributors, aggregated to the project level. From the same data, we took the reported number of individuals who were treated with ivermectin during mass treatment (File S1) and calculated the average therapeutic coverage of mass treatment in each project per calendar year (i.e., the fraction of the population at risk that was treated). Based on data from extensive pre-control mapping studies, we estimated the fraction of the population in the different endemicity categories and the mean pre-control infection level in each endemicity category (File S1).

For the years 2011–2015, we assumed that population size will increase according to the latest known national growth rate (as reported by the United Nations World Population Prospects, published 11 May 2010, accessed 24 October 2011). If therapeutic coverage in 2010 was already at or above 75%, we assumed that coverage in the years 2011–2015 will remain equal to that in 2010. For those few project in which this was not yet the case, we assumed that between 2011 and 2015, therapeutic coverage will be scaled up by 10 percentage points per year (conservative compared to reported coverage patterns in projects that started mass treatment between 1995 and 2010), to a maximum of 75% (conservative compared to the longest-running projects that reported stable coverage levels around 80% in 2008–2010).

### Simulating trends in infection and morbidity

For each unit of analysis (project, onchocerciasis type, endemicity), we simulated trends in infection, morbidity, and mortality in the ONCHOSIM model [6]–[8], considering the project-specific history of mass treatment (File S1). For each endemicity stratum, ONCHOSIM was calibrated so that it could reproduce the average pre-control level of infection (File S1). Furthermore, ONCHOSIM was calibrated to reproduce the association between the prevalence of infection and morbidity (visual impairment, blindness, and itch) as estimated by analysis of literature data (File S1). Based on previous studies with ONCHOSIM, we assumed that ivermectin instantly kills all microfilariae and permanently reduces the capacity of adult female worms to release microfilariae by 35% in treated individuals (with cumulative effects for repeated treatments) [4], [7]. Individual participation in mass treatment was assumed to depend on age, sex (pregnant women and children under the age of five were assumed to be excluded from treatment), random non-compliance (i.e., temporal factors), and systematic non-compliance (i.e., fixed individual factors other than age and sex e.g. inclination towards participation). Systematic non-compliance was assumed to play a larger role when overall treatment coverage was lower (i.e. when there is lower inclination to participate in general), and vice versa [6], [8]. No simulations were performed for hypoendemic areas, as ONCHOSIM predicts that transmission of infection is unsustainable without migration of infected flies and/or human, and information on migration was lacking. Instead, we assumed that the prevalence of infection and morbidity in hypoendemic areas was 1/3 of that in mesoendemic areas, both pre-control and during control. For non-endemic areas, we assumed that prevalence of infection and morbidity was always zero.

### Calculating the health impact

We combined the predicted trends in prevalence of infection, morbidity, and mortality with information on the number of people at risk, yielding an estimate of the absolute number of cases of infection, morbidity, and deaths in each stratum. After aggregation of these results over all APOC projects, we calculated the burden of disease in terms of disability-adjusted life years (DALYs), which in our case is the sum of years lived in disability due to troublesome itch, visual impairment, and blindness, weighted by the loss of quality of life due to each symptom: 0.068, 0.282, and 0.594, respectively [9]; and years of life lost due to excess mortality from blindness (File S1). Every incident case of blindness was attributed 8 years of life lost, based on the average age of onset of blindness in ONCHOSIM, the associated life-expectancy (16 years) of a healthy person of the same age, and an estimated 50% reduction in remaining life-expectancy due to blindness (File S1). The estimated annual burden of disease was compared to the burden in a counterfactual scenario in which the pre-control prevalence of infection and morbidity did not change (i.e., as if there were no mass treatment), yielding an estimate of the averted disease burden. All DALY estimates in the present study are undiscounted.

### Sensitivity analysis

We assessed the influence of uncertain model assumptions on the estimated health impact, by means of univariate and multivariate sensitivity analyses (File S1). In a univariate sensitivity analyses, we assumed extreme, though plausible parameter values for each of the selected parameters. In a multivariate sensitivity analysis, the analysis was repeated, based on 200 sets of random parameter values. Parameter values were randomly drawn from triangular distributions with modes equal to the values used in the main analysis, and minimum and maximum values equal to those used in the univariate sensitivity analyses. To arrive at a crude estimate of the uncertainty in the estimated health impact, the results of the multivariate sensitivity analysis were expressed as the 2.5 and 97.5 percentiles of results from 200 repeated analyses.

### Estimating the cost of APOC

We estimated the financial costs for coordination of ivermectin mass treatment taken on by APOC and national onchocerciasis task forces (beneficiary governments and non-governmental development organizations), based on APOC financial reports for The World Bank, which acts as fiscal agent for APOC. Because governments of beneficiary countries will eventually have to finance and coordinate ivermectin mass treatment, costs were estimated from a program perspective, not accounting for community costs and costs of donated drugs. For the years 1995–2003 and 2010, cost data for national onchocerciasis task forces were not available and were assumed to be proportional to APOC expenditures by a factor based on data available for other years. Expenditures for 2011–2015 were estimated based on the expected number of treatments in that period multiplied by the estimated cost per treatment in 2010. All costs are reported in nominal values, by which we mean that the presented costs are the amounts that were actually spent (i.e. uncorrected for inflation, and undiscounted).

## Results

**Figure 1:**
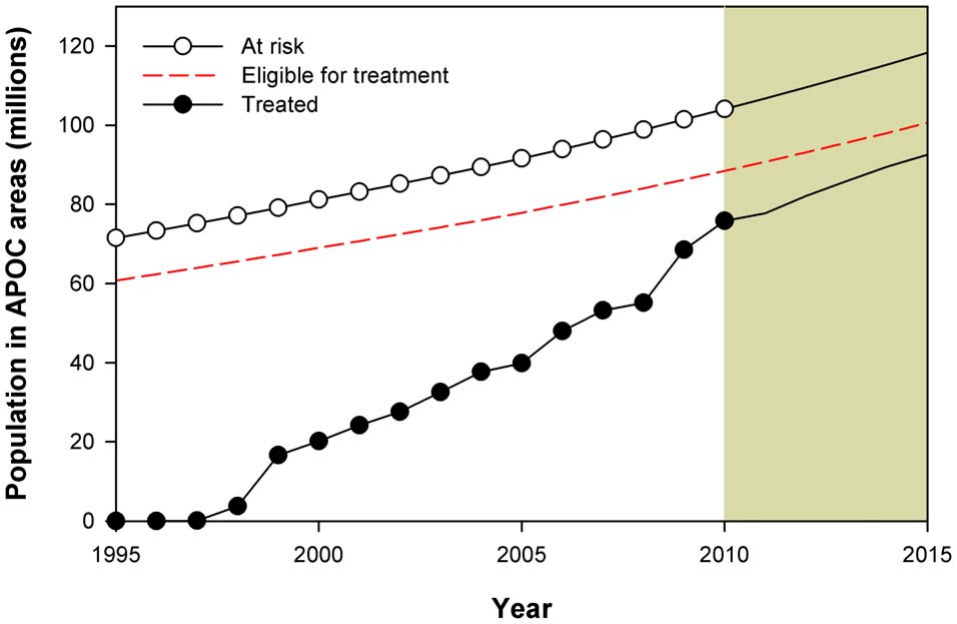
Population at risk and treated in areas covered by the African Programme for Onchocerciasis Control. Dots represent time points for which data were available; projections for 2011–2015 (shaded area) are based on the assumptions that populations continue to grow according to the latest known growth rates and that all projects scale up therapeutic coverage by 10 percentage points per year (up to a maximum coverage of 75%).

In 1995, the total population size in the APOC target area was 71.5 million (Figure 1), with 30% of the APOC target population living in hyperendemic communities, 31% in mesoendemic communities, 38% in hypoendemic communities surrounded by mesoendemic or hyperendemic areas, and 1% living in non-endemic communities. About 30% of the APOC population lived in savanna areas and 70% in forest or forest–savanna mosaic areas (Table 1). Before the inception of APOC in 1995, about 32 million people (45%) in APOC areas were infected with onchocerciasis, with 404,000 people (0.6%) blind because of onchocerciasis, another 889,000 (1.2%) suffering from visual impairment, and 10 million people (14%) suffering from troublesome itch. In the same year, a total of 1.6 million DALYs (22.8 DALYs per 1,000 persons) were lost due to onchocerciasis: 694,000 because of troublesome itch, 684,000 from blindness, and 251,000 due to visual impairment.

**Table 1:** Size and distribution of population in APOC target areas (thousands and fraction of total).

		Number of treatment rounds provided through 2010									
Onchocerciasis type	Endemicity class	1–2		3–5		6–9		10–13		Total	
Forest/mixed	Non-endemic	3	0.0%	129	0.1%	119	0.1%	342	0.3%	593	0. 6%
Forest/mixed	Hypoendemic	155	0.1%	5,669	5.4%	5,245	5.0%	14,170	13.6%	25,239	24.3%
Forest/mixed	Mesoendemic	71	0.1%	4,179	4.0%	4,210	4.0%	11,768	11.3%	20,228	19.4%
Forest/mixed	Hyperendemic	13	0.0%	4,128	4.0%	5,428	5.2%	15,201	14.6%	24,770	23.8%
**Forest/mixed**	**Total**	**243**	**0.2%**	**14,104**	**13.6%**	**15,002**	**14.4%**	**41,481**	**39.9%**	**70,831**	**68.1%**
Savanna	Non-endemic	0	0.0%	1	0.0%	1	0.0%	18	0.0%	19	0.0%
Savanna	Hypoendemic	0	0.0%	871	0.8%	1,048	1.0%	12,837	12.3%	14,756	14.2%
Savanna	Mesoendemic	0	0.0%	1,695	1.6%	1,143	1.1%	9,402	9.0%	12,240	11.8%
Savanna	Hyperendemic	0	0.0%	1,900	1.8%	255	0.2%	4,049	3.9%	6,203	6.0%
**Savanna**	**Total**	**0**	**0.0%**	**4,467**	**4.3%**	**2,446**	**2.4%**	**26,306**	**25.3%**	**33,219**	**31.9%**
**Grand Total**		**243**	**0.2%**	**18,571**	**17.8%**	**17,449**	**16.8%**	**67,787**	**65.1%**	**104,050**	**100.0%**

Populations were stratified by onchocerciasis type, endemicity class and the history of mass treatment. The history of mass treatment is expressed as the number of treatment rounds provided through 2010.

**Figure 2:**
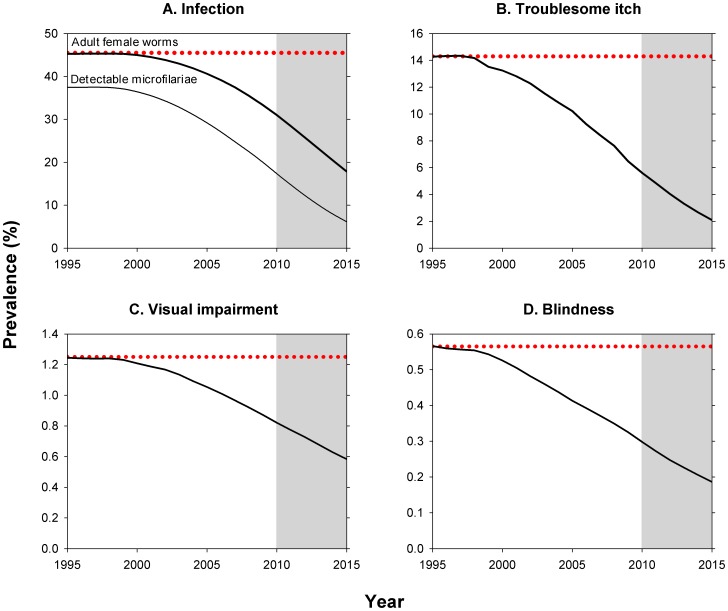
Predicted prevalence of onchocercal infection and morbidity in APOC areas from 1995 to 2015. Please note the different scales for the y-axes in the four panels. Shaded areas represent projections for 2011–2015. A) Prevalence of infection is defined as infestation with at least one adult female worm, or alternatively, presence of detectable microfilariae in the skin. B) Prevalence of troublesome itch, caused by onchocerciasis. C) Prevalence of onchocercal visual impairment, defined as corrected visual acuity (i.e. measured with glasses on or through pinhole) of <18/60 and ≥3/60 in the better eye. D) Prevalence of onchocercal blindness, defined as corrected visual acuity (i.e. measured with glasses on or through pinhole) of <3/60 or restriction of visual field to less than 10° in the better eye.

Mass treatment effectively started in 1997 (80,000 treatments) and was scaled up over the years, reaching an overall therapeutic coverage of about 73% in 2010 (75.8 million treatments; Figure 1). We estimated that the therapeutic coverage will increase to 78% by 2015 (92.5 million treatments). By 2010, about 65% of the population lived in areas subjected to 10–13 rounds of mass treatment, 17% in areas subjected to 6–9 rounds of mass treatment, 18% in areas subjected to 3–5 rounds of mass treatment, and less than 1% in areas subjected to only 1–2 rounds of mass treatment (Table 1). Cumulatively, about 500 million treatments with ivermectin were given between 1995 and 2010, with another 430 million expected to follow in the period 2011–2015. Considering the differences between projects in start year and patterns of scaling up of mass treatment, the prevalence of infection for APOC as a whole declined gradually and non-linearly over time, from 45% in 1995 to 31% in 2010, and to 18% in 2015 (Figure 2). Similarly, the prevalence of troublesome itch was reduced from 14% to 6% to 2%, and prevalence of visual impairment was reduced from 1.2% to 0.8% to 0.6%. Because of excess mortality among the blind and the fact that ivermectin prevented blindness in individuals who were already visually impaired, the prevalence of blindness declined more rapidly than that of visual impairment: from 0.6% to 0.3% to 0.2%.

**Figure 3:**
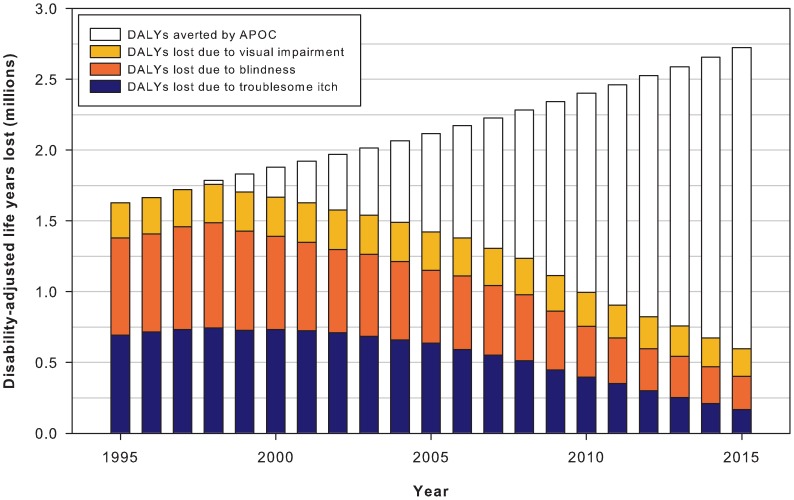
Disability-adjusted life years (DALYs) lost due to onchocerciasis from 1995 to 2015. The total height of the bars (colored plus blank) represents the estimated number of DALYs lost in a counterfactual scenario without ivermectin mass treatment (increasing trend due to population growth). The colored part of each bar represents the estimated actual number of DALYs lost (declining trend due to ivermectin mass treatment). The blank part of each bar therefore represents the annual number of DALYs averted by ivermectin mass treatment in the total APOC population.

In the counterfactual scenario without mass treatment, in which levels of infection and morbidity were stable, the absolute number of DALYs lost due to onchocerciasis would have increased over the years with population growth. In contrast, in the scenario that considers mass treatment with ivermectin, the absolute number of DALYs lost was predicted to decrease over the years. Due to these divergent trends, the number of DALYs averted by mass treatment with ivermectin was predicted to increase year by year (Figure 3). Overall, mass treatment with ivermectin averted 8.2 million DALYs between 1995 and 2010 (3.2 million due to itch, 4.4 million due to blindness, 0.6 million due to visual impairment). Moreover, we expect that APOC will avert another 9.2 million DALYs in the period 2011–2015, adding up to an expected total of 17.4 million averted DALYs by 2015 (Table 2). In relative terms, the disease burden of onchocerciasis was reduced from 22.8 DALYs per 1,000 persons in 1995 to 9.6 DALYs per 1,000 persons in 2010, and is expected to be further reduced to 5.0 DALYs per 1,000 persons by 2015.

**Table 2:** Health impact and cost of ivermectin mass treatment, 1995–2015.

Year	Health impact in number of DALYs averted (millions)	Costs for coordination of mass treatment (million US$)		
		APOC	National onchocerciasis task forces*	Total**
1995	0.00	0.0	0.0	0.0
1996	0.00	2.4	1.1	3.6
1997	0.00	2.4	1.1	3.6
1998	0.03	9.3	4.4	13.7
1999	0.13	9.3	4.4	13.7
2000	0.21	9.2	4.3	13.5
2001	0.29	9.2	4.3	13.5
2002	0.39	9.1	4.3	13.3
2003	0.47	11.3	5.3	16.7
2004	0.58	12.6	5.1	17.8
2005	0.69	13.5	4.0	17.6
2006	0.79	11.0	6.0	17.0
2007	0.92	13.7	7.7	21.4
2008	1.05	13.7	7.5	21.3
2009	1.23	21.2	10.0	31.1
2010	1.41	26.7	12.5	39.2
2011	1.56			40.2
2012	1.70			42.5
2013	1.83			44.4
2014	1.98			46.3
2015	2.13			47.9
**Subtotal 1995–2010**	**8.20**	**174.8**	**82.1**	**256.9**
**Total 1995–2015**	**17.39**			**478.1**

The health impact is expressed as the annual number of DALYs averted. Costs include those taken on by the African Programme for Onchocerciasis Control (APOC) and national onchocerciasis task forces (including beneficiary governments and non-governmental development organizations). All costs are expressed in nominal US$ (i.e., uncorrected for inflation and undiscounted), and do not include cost of donated drugs or government salaries.

* National onchocerciasis task force expenditures for the years 1995–2003 and 2010 were unknown; they were assumed to be equal to 47% of APOC expenditures, based on known expenditures for the years 2004–2009.

** Expenditures for 2011–2015 were estimated based on the expected number of treatments in that period multiplied by the estimated cost per treatment in 2010 ($0.52).

**Figure 4:**
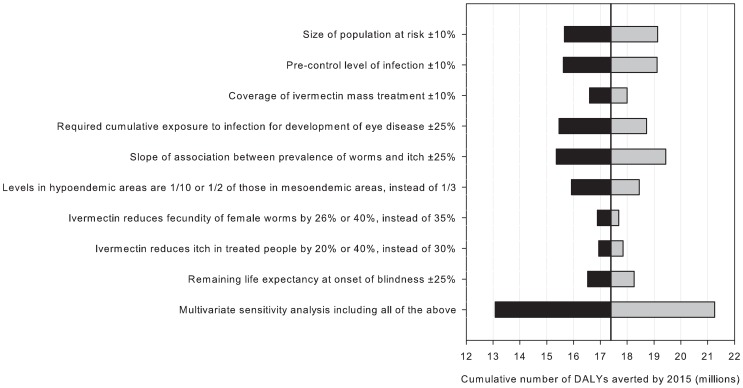
Sensitivity analysis for the estimated cumulative number of DALYs averted by 2015. The multivariate sensitivity analysis (last item) consisted of 200 repeated analyses, based on 200 sets of random parameter values, which were drawn from triangular distributions with modes equal to parameter values used in the main analysis, and minimum and maximum values equal to parameter values used in the univariate sensitivity analysis (first eight items of this figure). The results of the multivariate sensitivity analysis are expressed as the 2.5 and 97.5 percentiles of results from 200 repeated analyses.

Univariate sensitivity analyses identified the following parameters as having the most influence on the estimated health impact: the population at risk, pre-control levels of infection, and the associations between infection and itch and eye disease (Figure 4). The multivariate sensitivity analysis showed that the estimated cumulative number of DALYs averted could be up to 25% higher or lower, when we considered the separate sources of uncertainty simultaneously (6.0–9.8 million DALYS cumulatively averted by 2010, and 13.1–21.3 million DALYs cumulatively averted by 2015; Figure 4).

Between 1995 and 2010, coordination of mass treatment cost roughly US$257 million (Table 2), of which US$175 million was disbursed by APOC and US$82 million by national onchocerciasis task forces (cost of donated drugs and government salaries not included). Assuming that costs will rise proportionally with the number of treatments, mass treatment was expected to cost another US$221 million between 2011 and 2015, adding up to a total cost of US$478 million by 2015.

## Discussion

We estimated the health impact and cost of mass treatment with ivermectin for the 20-year period that APOC is scheduled to run as a morbidity control program (1995–2015). Our simulations suggest that mass treatment with ivermectin has markedly reduced the prevalence of infection with *O. volvulus*, troublesome itch, visual impairment, and blindness in APOC areas, averting an estimated 8.2 million DALYs due to onchocerciasis by 2010 at a nominal financial cost of about US$257 million (excluding cost of donated drugs). We expect that APOC will avert another 9.2 million DALYs between 2011 and 2015, at a nominal financial cost of US$221 million.

Our estimate of APOC's health impact only considered eye disease and troublesome itch, and would be even higher if other clinical manifestations of onchocerciasis would have been taken into account. For instance, disfiguring skin disease also contributes to the disease burden of onchocerciasis and is known to be reduced by ivermectin [10]–[13]. Further, epilepsy may be associated with onchocerciasis, as suggested by a growing but still uncertain base of evidence [14]. However, we chose to include only the most important disease manifestations for which data were available for model calibration (i.e., eye disease and troublesome itch). Furthermore, we did not include the effect of ivermectin on diseases that are co-endemic with onchocerciasis, such as soil transmitted helminthiases, ectoparasitic infections, and lymphatic filariasis [15]. Other minor factors leading to an underestimation of the health impact are that we only considered the effect of ivermectin on the capacity of adult female worms to release microfilariae and its microfilaricidal effect, whereas ivermectin may additionally have a modest effect on adult worm viability [16], [17]. Furthermore, we ignored between-village variation in coverage, which is perhaps most extreme in the phase of scaling up: in some projects, treatment started in a subpopulation with high coverage, while the other part of the population did not yet receive mass treatment (which is more efficient than treating the entire project population at an equivalent average coverage). We may have somewhat overestimated the number of life years lost due to excess mortality from blindness during and after mass treatment, causing a small underestimation in the number of DALYs averted. This is because we appointed a fixed number of life years lost to every new case of blindness, while regular ivermectin treatment is expected to postpone the onset of blindness to a higher age, reducing the number of life years lost due to blindness. Furthermore, we did not consider a possible association between excess mortality and (high) microfilarial load [18], [19].

There are several factors that may (partly) counterweigh the underestimation of the health impact of APOC described above. Therapeutic coverage may have been over-reported by community members responsible for the distribution of ivermectin, either because of incomplete estimates of the community population or to inflate their own performance. Yet, the estimated health impact of APOC by 2015 would decrease by only 0.8 million averted DALYs if we assume that coverage were to be systematically 10% lower than reported. Also, we ignored any mass treatment prior to the inception of APOC, whereas in reality, ivermectin distribution had already started in a limited number of foci (here morbidity levels had already been reduced somewhat, but not on account of APOC). Taking all above sources of under- and over-estimation into account, we believe that the true health impact of APOC is still slightly higher than our calculations.

The validity of our results, as in any simulation study, depends on the quality of the model and its assumptions. ONCHOSIM was first developed in the early nineties and has earned trust over the years from the large scale control programs. ONCHOSIM has been used to successfully mimic observed epidemiological data from various locations [4], [20]–[22], and has been used for policy making in the West-African Onchocerciasis Control Programme [7]. Efforts to validate the model continue. We have recently compared ONCHOSIM predictions to longitudinal data from Senegal and Gambia [23] and found that model-predicted trends in mf prevalence during 14 to 16 years of mass treatment were broadly consistent with the observed trends, although the mf prevalence sometimes seemed to decline slightly faster than predicted (unpublished data). Furthermore, our model predictions for trends in itch were comparable to the reported average trend in APOC sentinel areas [13]; after five to six years of mass treatment at 70–80% coverage, itch prevalence was reported to decline from 16% to 7%, and we predicted a decline from 14% to 6.5% for areas with similar pre-control levels of infection and history of mass treatment. Likewise, our model adequately reproduced trends in onchocercal blindness during vector control in West Africa (File S1). Although the above suggests that our model predictions are realistic, our estimates remain subject to uncertainty and it would be good to have them confirmed by more field data, especially regarding trends in morbidity during mass treatment.

Even though the model seems to be reliable, we should consider potentially important sources of uncertainty in our analysis. An often debated factor concerns the effect of ivermectin on adult worms. The univariate sensitivity analysis showed that the assumed treatment effects of ivermectin on the capacity of adult worms to release microfilariae influenced the estimated health impact only marginally. We did not study the effects of assuming no cumulative effects of ivermectin on worm fecundity, whereas it has been suggested that the latter may be the case [24]. However, if we had, ivermectin efficacy parameters would have been calibrated such that the model-predicted trends in mf prevalence and density were still in agreement with observed trends [4], [22], and therefore predicted trends in infection levels and morbidity should not have differed much from the current model's predictions. The sensitivity analysis showed that alternative assumptions for the effect of ivermectin on itch (the only reversible symptom under consideration) also influenced the estimated health impact only marginally. The most influential assumptions in our analysis were related to the estimated size of the population at risk, pre-control levels of infection, and the assumed associations between infection and morbidity, which were all based on data. Even though the multivariate sensitivity analysis suggested considerable overall uncertainty in our estimate of the health impact (±25%), the magnitude of the predicted impact was always large.

With an estimated 8.2 million DALYs averted in a 15-year period and a predicted doubling in the subsequent 5 years, the predicted health impact of APOC is impressive. According to our calculations, mass treatment against onchocerciasis cost about a nominal US$31 per undiscounted DALY averted between 1995 and 2010. According to World Health Organization guidelines [25], this is highly cost-effective, as it is below the per capita gross domestic product of most countries covered by APOC (27–1,545 international dollar per capita; Global Health Observatory Data Repository, accessed 2 August 2012). Furthermore, this cost-effectiveness is comparable to or even better than those for several other public health interventions. For example, the life-time cost-effectiveness of prophylaxis against mother-to-child transmission of HIV in a resource-limited setting has been estimated at US$52 per undiscounted DALY (incremental cost-effectiveness ratio of World Health Organization guidelines versus minimal standard of care) [26]. The cost-effectiveness of large-scale, long-term (30-year period) public health interventions targeting other neglected tropical diseases has been estimated at US$4–US$29 per DALY (mass drug administration against lymphatic filariasis), US$38 per DALY (case detection and treatment for leprosy), US$260 per DALY (vector control against Chagas disease), and US$48–US$303 (vector control against lymphatic filariasis) [27]. Mass treatment against onchocerciasis is of even better value (US$27 per DALY) if expected health gains and costs for the period 2011–2015 are included. In view of the anticipated elimination of infection so that mass treatment can be stopped altogether, the cost-effectiveness will be even better than our calculations suggest [23].

The objective of APOC is to establish country-led systems for onchocerciasis control by 2015, which means that countries and their partners will have to carry full financial responsibility by that year. Our results indicate that cost per treatment with ivermectin in APOC areas is affordable (US$0.51 per treatment, excluding cost of donated drugs) and comparable to the costs of existing national mass treatment programs for the elimination of lymphatic filariasis (US$0.06–US$2.23 per treatment) [28]. Mass treatment with ivermectin, however, also involves costs for society not covered by the program. From published data for two Nigerian communities, we derived that these costs are about US$0.23 per treatment (excluding start-up costs) [29]. Based on this estimate, the sum of program and community costs for mass treatment with ivermectin was approximately US$370 million from 1995 to 2010 and will be another US$320 million for 2011–2015. In addition to costs, there are significant benefits for society that countries need to take into account, such as prevented productivity losses resulting from blindness and itch. Blindness in rural Africa has previously been assumed to result in an annual productivity loss of US$150 per case [30]. Likewise, the productivity loss due to itch among coffee plantation workers in an Ethiopian site has been estimated at around US$5.32 per month per case [12]. Combined with our predictions of health impact, these figures suggest that by 2015, APOC will have averted a staggering US$2.2 billion due to productivity losses from blindness (US$517 million) and itch (US$1.7 billion, assuming productivity losses in 25% of people with itch). In other words, beneficiary countries should expect economic benefit from mass treatment that outweighs any costs.

Clearly, all of the above calculations apply only under the condition that countries do not themselves pay for the drug ivermectin. The amount of ivermectin donated up to 2010 represents a value of US$2.1 billion, assuming 2.8 tablets per treatment and a commercial price per tablet of US$1.50 plus US$0.005 shipping costs (personal communication with Dr. A. Hopkins, director of the Mectizan Donation Program). This amount is eight times the program costs for coordinating mass treatment. Likewise, for the period 2011–2015, the value of donated ivermectin will be an additional US$1.8 billion. Therefore, mass treatment with ivermectin can be sustained only with donation of ivermectin, which Merck has pledged to continue for as long as necessary.

We expect that levels of infection in the APOC target area will have fallen drastically by 2015 (overall prevalence of adult female worms 18%). The implication is that by that time, transmission of infection may be almost interrupted in areas with favorable conditions for elimination, such as high coverage of mass treatment, sufficient treatment rounds, and/or low to medium pre-control levels of infection [31]. Until recently, elimination of onchocerciasis from Africa was thought to be impossible by means of mass treatment alone, considering the large size of the transmission zones, mobility of the vectors and human populations, and poor compliance with mass treatment [32]. Following reports of elimination of onchocerciasis from foci in Mali and Senegal by mass treatment alone [23], however, interest has renewed in elimination of onchocerciasis from Africa [33]. Following this, WHO has recently been advised to extend APOC mandate by ten years to 2025 with the new aim of eliminating infection with *O. volvulus*, where possible. With this new motivation, we may indeed expect focal elimination of infection, resulting in even more health gains from mass treatment with ivermectin in the future and the possibility of being able to end mass treatment altogether.

According to our simulations, APOC has had a remarkable impact on population health in Africa between 1995 and 2010. This health impact is expected to double during the subsequent five years. Further, APOC is a highly cost-effective public health programs, and given the anticipated elimination of onchocerciasis from APOC areas, we expect even more health gains and a more profitable cost-effectiveness of mass treatment with ivermectin in the near future. Our study fully supports the advice to continue APOC activities for another ten years.

## Supporting Information

File S1 This document describes the details of the methods that were used in this study, both in text and figures. This supplement is divided into appropriately named sections, which are intended to be read when referred to from the corresponding methods section of the main article (i.e. the supplement is not intended to be read as a stand-alone document).
(PDF)

## References

[1] Duke BO (1993) The population dynamics of Onchocerca volvulus in the human host. Trop Med Parasitol 44: 61–68. 8367667

[2] Boatin B (2008) The Onchocerciasis Control Programme in West Africa (OCP). Ann Trop Med Parasitol 102 Suppl 1: 13–17. 18718148 10.1179/136485908X337427

[3] Noma M, Nwoke BE, Nutall I, Tambala PA, Enyong P, et al (2002) Rapid epidemiological mapping of onchocerciasis (REMO): its application by the African Programme for Onchocerciasis Control (APOC). Ann Trop Med Parasitol 96 Suppl 1: S29–39. 12081248 10.1179/000349802125000637

[4] Plaisier AP, Alley ES, Boatin BA, Van Oortmarssen GJ, Remme H, et al (1995) Irreversible effects of ivermectin on adult parasites in onchocerciasis patients in the Onchocerciasis Control Programme in West Africa. J Infect Dis 172: 204–210. 7797912 10.1093/infdis/172.1.204

[5] Basáñez MG, Pion SD, Boakes E, Filipe JA, Churcher TS, et al (2008) Effect of single-dose ivermectin on Onchocerca volvulus: a systematic review and meta-analysis. Lancet Infect Dis 8: 310–322. 18471776 10.1016/S1473-3099(08)70099-9

[6] Plaisier AP, van Oortmarssen GJ, Habbema JD, Remme J, Alley ES (1990) ONCHOSIM: a model and computer simulation program for the transmission and control of onchocerciasis. Comput Methods Programs Biomed 31: 43–56. 2311368 10.1016/0169-2607(90)90030-d

[7] Plaisier AP, Alley ES, van Oortmarssen GJ, Boatin BA, Habbema JD (1997) Required duration of combined annual ivermectin treatment and vector control in the Onchocerciasis Control Programme in west Africa. Bull World Health Organ 75: 237–245. 9277011 PMC2486951

[8] Habbema JDF, Oostmarssen GJ, Plaisier AP (1996) The ONCHOSIM model and its use in decision support for river blindness control. In: Isham V, Medley G, editors. Models for infectious human diseases - their stucture and relation to data. Cambridge: Cambridge Universitry Press. pp. 360–380.

[9] World Health Organization (2004) Global Burden of Disease update 2004: disability weights for diseases and conditions. Geneva: World Health Organization.

[10] Murdoch ME, Asuzu MC, Hagan M, Makunde WH, Ngoumou P, et al (2002) Onchocerciasis: the clinical and epidemiological burden of skin disease in Africa. Ann Trop Med Parasitol 96: 283–296. 12061975 10.1179/000349802125000826

[11] Alonso LM, Murdoch ME, Jofre-Bonet M (2009) Psycho-social and economical evaluation of onchocerciasis: a literature review. Soc Med 4: 8–31.

[12] Kim A (1997) Health and Labor Productivity: the economic impact of onchocercal skin disease. The World Bank

[13] Ozoh GA, Murdoch ME, Bissek AC, Hagan M, Ogbuagu K, et al (2011) The African Programme for Onchocerciasis Control: impact on onchocercal skin disease. Trop Med Int Health 16: 875–883. 21481109 10.1111/j.1365-3156.2011.02783.x

[14] Pion SD, Kaiser C, Boutros-Toni F, Cournil A, Taylor MM, et al (2009) Epilepsy in onchocerciasis endemic areas: systematic review and meta-analysis of population-based surveys. PLoS Negl Trop Dis 3: e461. 19529767 10.1371/journal.pntd.0000461 PMC2691484

[15] Tielsch JM, Beeche A (2004) Impact of ivermectin on illness and disability associated with onchocerciasis. Trop Med Int Health 9: A45–56. 15078278 10.1111/j.1365-3156.2004.01213.x

[16] Awadzi K, Attah SK, Addy ET, Opoku NO, Quartey BT (1999) The effects of high-dose ivermectin regimens on Onchocerca volvulus in onchocerciasis patients. Trans R Soc Trop Med Hyg 93: 189–194. 10450448 10.1016/s0035-9203(99)90305-x

[17] Gardon J, Boussinesq M, Kamgno J, Gardon-Wendel N, Demanga N, et al (2002) Effects of standard and high doses of ivermectin on adult worms of Onchocerca volvulus: a randomised controlled trial. Lancet 360: 203–210. 12133654 10.1016/S0140-6736(02)09456-4

[18] Little MP, Breitling LP, Basáñez MG, Alley ES, Boatin BA (2004) Association between microfilarial load and excess mortality in onchocerciasis: an epidemiological study. Lancet 363: 1514–1521. 15135599 10.1016/S0140-6736(04)16151-5

[19] Walker M, Little MP, Wagner KS, Soumbey-Alley EW, Boatin BA, et al (2012) Density-dependent mortality of the human host in onchocerciasis: relationships between microfilarial load and excess mortality. PLoS Negl Trop Dis 6: e1578. 22479660 10.1371/journal.pntd.0001578 PMC3313942

[20] Plaisier AP, van Oortmarssen GJ, Remme J, Alley ES, Habbema JD (1991) The risk and dynamics of onchocerciasis recrudescence after cessation of vector control. Bull World Health Organ 69: 169–178. 1860147 PMC2393088

[21] Plaisier AP, van Oortmarssen GJ, Remme J, Habbema JD (1991) The reproductive lifespan of Onchocerca volvulus in West African savanna. Acta Trop 48: 271–284. 1674401 10.1016/0001-706x(91)90015-c

[22] Alley ES, Plaisier AP, Boatin BA, Dadzie KY, Remme J, et al (1994) The impact of five years of annual ivermectin treatment on skin microfilarial loads in the onchocerciasis focus of Asubende, Ghana. Trans R Soc Trop Med Hyg 88: 581–584. 7992347 10.1016/0035-9203(94)90172-4

[23] Diawara L, Traore MO, Badji A, Bissan Y, Doumbia K, et al (2009) Feasibility of onchocerciasis elimination with ivermectin treatment in endemic foci in Africa: first evidence from studies in Mali and Senegal. PLoS Negl Trop Dis 3: e497. 19621091 10.1371/journal.pntd.0000497 PMC2710500

[24] Bottomley C, Isham V, Collins RC, Basáñez MG (2008) Rates of microfilarial production by Onchocerca volvulus are not cumulatively reduced by multiple ivermectin treatments. Parasitology 1–11. 18831801 10.1017/S0031182008000425

[25] WHO Commission on Macroeconomics and Health (2001) Macroeconomics and health: investing in health for economic development. Report of the Commision on Macroeconomics and Health. Geneva: World Health Organization.

[26] Shah M, Johns B, Abimiku A, Walker DG (2011) Cost-effectiveness of new WHO recommendations for prevention of mother-to-child transmission of HIV in a resource-limited setting. AIDS 25: 1093–1102. 21505317 10.1097/QAD.0b013e32834670b9

[27] Remme JHF, Feenstra P, Lever PR, Médici A, Morel C, et al. (2006) Tropical Diseases Targeted for Elimination: Chagas Disease, Lymphatic Filariasis, Onchocerciasis, and Leprosy. In: Jamison DT, Breman JG, Measham AR, Alleyne G, Claeson M et al.., editors. Disease Control Priorities in Developing Countries (2nd Ed). Washington DC: The World Bank/Oxford University Press. 21250324

[28] Goldman AS, Guisinger VH, Aikins M, Amarillo ML, Belizario VY, et al (2007) National mass drug administration costs for lymphatic filariasis elimination. PLoS Negl Trop Dis 1: e67. 17989784 10.1371/journal.pntd.0000067 PMC2041814

[29] Onwujekwe O, Chima R, Shu E, Okonkwo P (2002) Community-directed treatment with ivermectin in two Nigerian communities: an analysis of first year start-up processes, costs and consequences. Health Policy 62: 31–51. 12151133 10.1016/s0168-8510(01)00226-3

[30] Benton B (1998) Economic impact of onchocerciasis control through the African Programme for Onchocerciasis Control: an overview. Ann Trop Med Parasitol 92 Suppl 1: S33–39. 9861265 10.1080/00034989859537

[31] Winnen M, Plaisier AP, Alley ES, Nagelkerke NJ, van Oortmarssen G, et al (2002) Can ivermectin mass treatments eliminate onchocerciasis in Africa? Bull World Health Organ 80: 384–391. 12077614 PMC2567795

[32] Dadzie Y, Neira M, Hopkins D (2003) Final report of the Conference on the eradicability of Onchocerciasis. Filaria J 2: 2. 12605722 10.1186/1475-2883-2-2 PMC150378

[33] Mackenzie CD, Homeida MM, Hopkins AD, Lawrence JC (2012) Elimination of onchocerciasis from Africa: possible? Trends Parasitol 28: 16–22. 22079526 10.1016/j.pt.2011.10.003

