# Supplementary material for: African Programme for Onchocerciasis Control 1995–2015: Model-Estimated Health Impact and Cost
Source: PLoS Negl Trop Dis. 2013 Jan 31;7(1):e2032. doi: 10.1371/journal.pntd.0002032 (PMC3561133; doi:10.1371/journal.pntd.0002032)
Supplement: File S1 — This document describes the details of the methods that were used in this study, both in text and figures. This supplement is divided into appropriately named sections, which are intended to be read when referred to from the corresponding methods section of the main article (i.e. the supplement is not intended to be read as a stand-alone document). (PDF) [file pntd.0002032.s001.pdf]

African Programme for Onchocerciasis Control 1995–2015: model-estimated health impact and cost – Supplement

Luc E. Coffeng[1], Wilma A. Stolk[1], Honorat G.M. Zouré[2], J. Lennert Veerman[1,3], Koffi B. Agblewonu[2], Michele E. Murdoch[4], Mounkaila Noma[2], Grace Fobi[2], Jan Hendrik Richardus[1], Donald A.P. Bundy[5], Dik Habbema[1], Sake J. de Vlas[1], Uche V. Amazigo[6]*

[1] Department of Public Health, Erasmus MC, University Medical Center Rotterdam, Rotterdam, The Netherlands
[2] African Programme for Onchocerciasis Control, Ouagadougou, Burkina Faso
[3] School of Population Health, The University of Queensland, Brisbane, Australia
[4] Department of Dermatology, Watford General Hospital, Watford, United Kingdom
[5] Human Development Network, The World Bank, Washington District of Columbia, United States of America
[6] Independent consultant, Enugu, Nigeria

*Correspondence to:
Luc Coffeng, Department of Public Health, Erasmus MC, University Medical Center Rotterdam, P.O. box 2040, 3000 CA Rotterdam, The Netherlands; l.coffeng@erasmusmc.nl, luccoffeng@gmail.com; +31 10 7038460

Contents

S1 Population at risk ... 3
S2 History of mass treatment with ivermectin in APOC ... 3
S3 Pre-control level of infection ... 3
S3.1 Categorization of project populations in endemicity categories ... 4
S3.2 Mean prevalence of infection per endemicity category ... 6
S4 Modeling trends in infection and morbidity in ONCHOSIM ... 6
S5 Calibration of ONCHOSIM parameters for transmission ... 7
S6 Calibration of ONCHOSIM parameters for eye disease ... 8
S6.1 Disease threshold and excess mortality for savanna type of onchocerciasis ... 8
S6.2 Disease threshold for forest/mixed type of onchocerciasis ... 10
S7 Calibration of model parameters for troublesome itch ... 11
S7.1 Statistical association between nodule prevalence and itch ... 12
S7.2 The association between prevalence of itch and adult female worms ... 13
S7.3 The effect of ivermectin on prevalence of itch ... 14
S8 Calculation of the burden of disease ... 15
S9 Sensitivity analysis ... 15
S10 Acknowledgements ... 16
References ... 18

S1 Estimating the population at risk

Information on the size of the population at risk for onchocerciasis in APOC projects areas was available per calendar year from APOC's mass treatment database. The population estimates in this database are based on annual village census figures as reported by individuals responsible for the distribution of ivermectin in the community, aggregated to the project level.

In most projects, mass treatment was implemented over the years, starting in some villages and expanding geographically over the years. Also, over the course of time, some projects were expanded to cover larger areas, based on updated estimates of the geographical distribution of infection. Because for some projects, the mass treatment data only holds population estimates for areas where mass treatment was actually implemented, we regarded the year with the largest estimate (standardized to 2010, based on national population growth rates as reported by the United Nations World Population Prospects, published 11 May 2010, accessed 24 October 2011) as the reference year with the best estimate of the population at risk for infection. We then estimated the 'true' population at risk for all other years, based on national growth rates. Population sizes for the period 2011–2015 were extrapolated from the population sizes for 2010, assuming that the growth rates for 2011–2015 are equal to that of 2010.

S2 Estimating the history of mass treatment with ivermectin in APOC

Information on the history of mass treatment with ivermectin was obtained from APOC's mass treatment database, which contains information by project and year about the number of people treated and the population size. In section S1, we already explained how we dealt with uncertainty in the data to estimate the total population of projects by year. Data on the number of persons treated were thought to be more robust, as these were reported by individuals responsible for distribution of ivermectin who are trained to observe people when they take ivermectin. Also, ivermectin distributors are retrained every year for at least 3 consecutive years, which should improve the robustness of the reported data. The therapeutic coverage, here defined as the fraction of the population that was treated with ivermectin, by year and project, was estimated by dividing the number of reported persons treated by the size of the target population (size based on the corrected estimate, described in S1). The calculated therapeutic coverage represents the average coverage in a project population in each year. We did not mimic between-village variation in coverage, which is perhaps most extreme in the phase of scaling up: in some projects, treatment started in a subpopulation with high coverage, while the other part of the population did not yet receive mass treatment (zero coverage). By taking the average, we may have been somewhat pessimistic about the impact of APOC, as low coverage in a large population is less effective than high coverage in a small population because of transmission effects. This may have been especially the case for situations where mass treatment started in the most highly endemic areas of a project.

S3 Estimating the pre-control level of infection

Because the effect of mass treatment with ivermectin depends on the pre-control level of infection in a population (aside from therapeutic coverage), the health impact of APOC was estimated for strata of population exposed to different pre-control levels of infection, a proxy for intensity of transmission. To do this, we first estimated the geographical distribution of pre-control levels of infection and divided project populations in endemicity categories so that we could model trends in infection and morbidity accordingly. For this exercise we defined four categories of pre-control nodule prevalence: non-endemic (nodule prevalence in adult males <1%), hypoendemic (≥1% and <20%), mesoendemic (≥20% and <40%), and

hyperendemic (≥ 40%). The geographical distribution of infection in each project was expressed as the fraction of the population living in each endemicity category (Table 1 in main manuscript). We assumed that these fractions (representing geographical areas) were stable during the period for which calculations were done (1995 to 2015). Next, for each endemicity category, the mean pre-control prevalence of infection was determined, serving as a starting point for ONCHOSIM simulations.

S3.1 Categorization of project populations in endemicity categories

The distribution of infection in a project was estimated from the database for Rapid Epidemiological Mapping of Onchocerciasis (REMO) [1,2]. These data were assumed to be representative for the geographical area covered by APOC, in terms of population size and level of infection. The REMO data have been gathered according to a strict protocol: surveys were started in a selection of villages perceived to be at high risk for onchocerciasis transmission (e.g. close to a major river in an area where blackflies are known to be present). In each selected village, a sample of 30 to 50 adult males (age 20 years and older) was examined for onchocercal nodules (henceforth referred to as nodule prevalence). For any village that proves at least mesoendemic (nodule prevalence ≥20%), a secondary village at least 10 km away was also surveyed. The cut-off nodule prevalence of ≥20% corresponds to an mf prevalence of ≥40% and was used as an indication of considerable risk for onchocercal blindness in a community [1,2].

Because the REMO data are based on samples of 30 to 50 individuals, there is a good chance that in low-endemic villages zero individuals with nodules are observed, and that in highly endemic villages all examined individuals have nodules (whereas the 'true' prevalence is not 0% or 100%). In other words, the sampling error at the village level introduced additional variation in observed geographic distribution of nodule prevalences. Therefore, the variation of the observed distribution of nodule prevalences overestimates the true geographic variation in nodule prevalences. Consequently, when using the frequency distribution of nodule prevalences within a project as a measure of the geographical distribution of infection, the fraction of the population in low-endemic and highly endemic areas is overestimated. This error can be circumvented by taking account of the sampling error at the village level.

We assumed that in the REMO data, the reported number of adult males with nodules in each village (k) is a sample from a binomial distribution Bin(n, p) with n equal to the number of observations and p representing the nodule prevalence among adult males. To circumvent the error described above, we furthermore assumed that for any given village p is unknown (i.e., p is not necessarily equal to k divided by n), and that the range of unknown nodule prevalences p within a project area follows a beta distribution Beta(α, β). This beta distribution then represents the 'true' but unobserved distribution of nodule prevalence in a project, and can be used to determine the fraction of the population in each endemicity category. For each project, we estimated the shape parameters α and β of the beta distribution with a beta-binomial regression model in R^+ (version 2.13.2, Vienna, Austria, 2011), using a maximum-likelihood approach (package *VGAM*) [3]. When no sample size n was available for a village in the REMO data, we assumed n equal to the median sample size of the other villages in the project area (usually ~30, only in Nigeria usually ~50). If the sample sizes were unknown for all villages in a project (four projects in Uganda), a sample size of 30 was assumed, as specified in the REMO protocol [1,2].

For each project we examined the dispersion of nodule prevalence within the project (a measure of heterogeneity, defined as $1 / (1+\alpha+\beta)$). As may be expected, the dispersion of nodule prevalence was higher in forest areas (Figure S1), probably because transmitting blackflies are restricted in their movement by dense forest, resulting in focally high prevalences of infection. Furthermore, dispersion of infection was associated with the mean

prevalence of infection in the project (α / (α+β)); dispersion was highest for levels of infection around 50% and was mostly lower for any other level of infection (Figure S1). Therefore, we standardized the dispersion of nodule prevalences over the whole APOC area by defining dispersion as a function of the mean nodule prevalence in a project. We used a linear regression model to predict the logit of the estimated dispersion within a project from the mean nodule prevalences in a project (Figure S1). We included both a linear and a square term for nodule prevalence, assuming that the dispersion would be lowest at very high and very low mean prevalences. This assumption was robust, as final estimates of the health impact were very similar when based on the means and dispersions of infection levels, estimated without the constraint of a quadratic association between the two. We also included a linear term for type of onchocerciasis, allowing for differences in geographical distribution of infection in savanna and forest areas. The linear regression parameters were estimated in R^+ (version 2.11.1, package *glm*), while weighting the data for the number of villages sampled per project (weight equal to square root of number of villages sampled in each project). Using this linear regression model, we re-estimated the dispersion of nodule prevalence in each project and calculated the final shape parameters of the beta distributions of nodule prevalence.

Finally, for each project we calculated the fraction of the populations in each of the previously mentioned endemicity categories, based on the cumulative beta distribution of nodule prevalence in adult males.

Figure S1: Nodule prevalence in adult males: mean and dispersion per project and onchocerciasis type (blue represents forest/mixed areas; red represents savanna areas), as estimated from the database for Rapid Epidemiological Mapping of Onchocerciasis. A data point represents a project; the size of a data point reflects the number of villages that was sampled in the project. The regression lines are based on a linear regression model that predicts the logit dispersion from the mean nodule prevalence in a project.

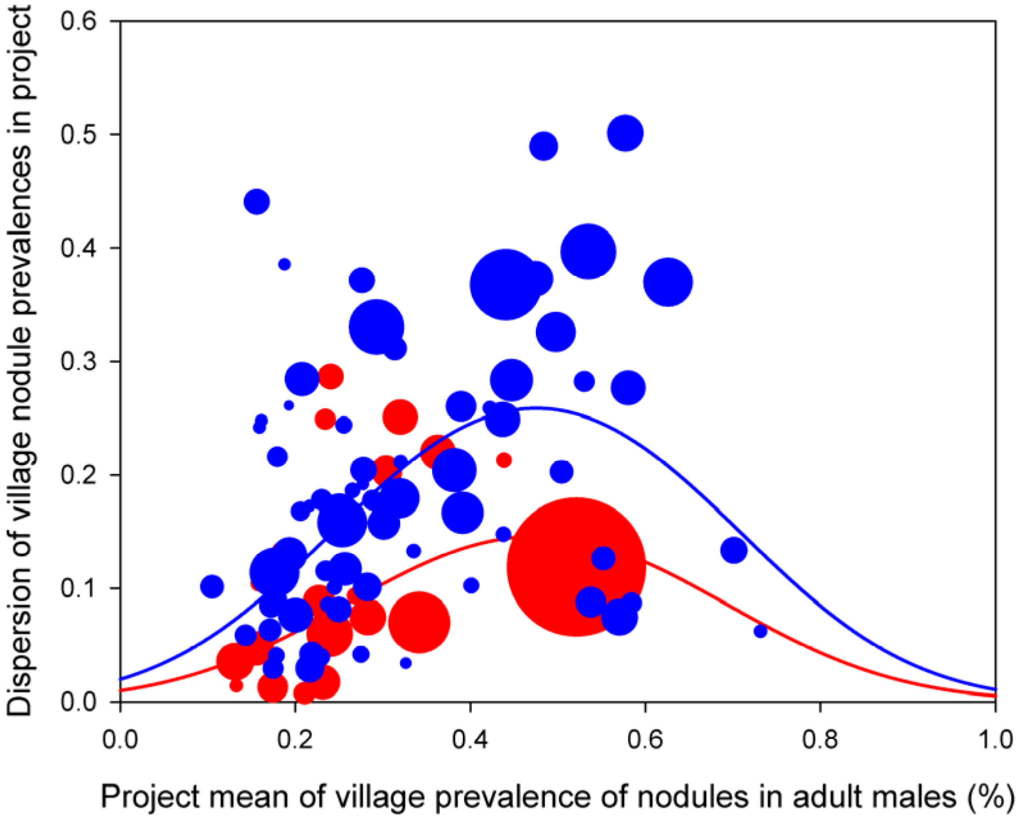

S3.2 Mean prevalence of infection per endemicity category

As explained above, we determined the mean pre-control nodule prevalence in each endemicity category to serve as a starting point for modeling trends in infection. To minimize variation due to the fact that for some projects relatively few villages were sampled, the mean pre-control nodule prevalence in each endemicity category was determined over the whole of APOC. This was done by taking 100,000 samples from the beta distribution of nodule prevalence for each project and dividing them in the aforementioned endemicity categories. Next, we calculated the overall mean nodule prevalence for each category over the whole of APOC, weighted for the size of the population in each project.

The mean nodule prevalence among adult males in mesoendemic areas was estimated at 29%; for hyperendemic areas, it was estimated at 61%. Because no simulations were performed for hypoendemic and non-endemic areas, there was no need to estimate the mean prevalence of infection for these categories.

Figure S2: The relationship between the prevalence of nodules in adult males and the prevalence of mf among the total population aged 5 years and older. The horizontal and vertical lines indicate the threshold values for the hypoendemic, mesoendemic and hyperendemic categories. Reproduced from a previous publication by Remme [4].

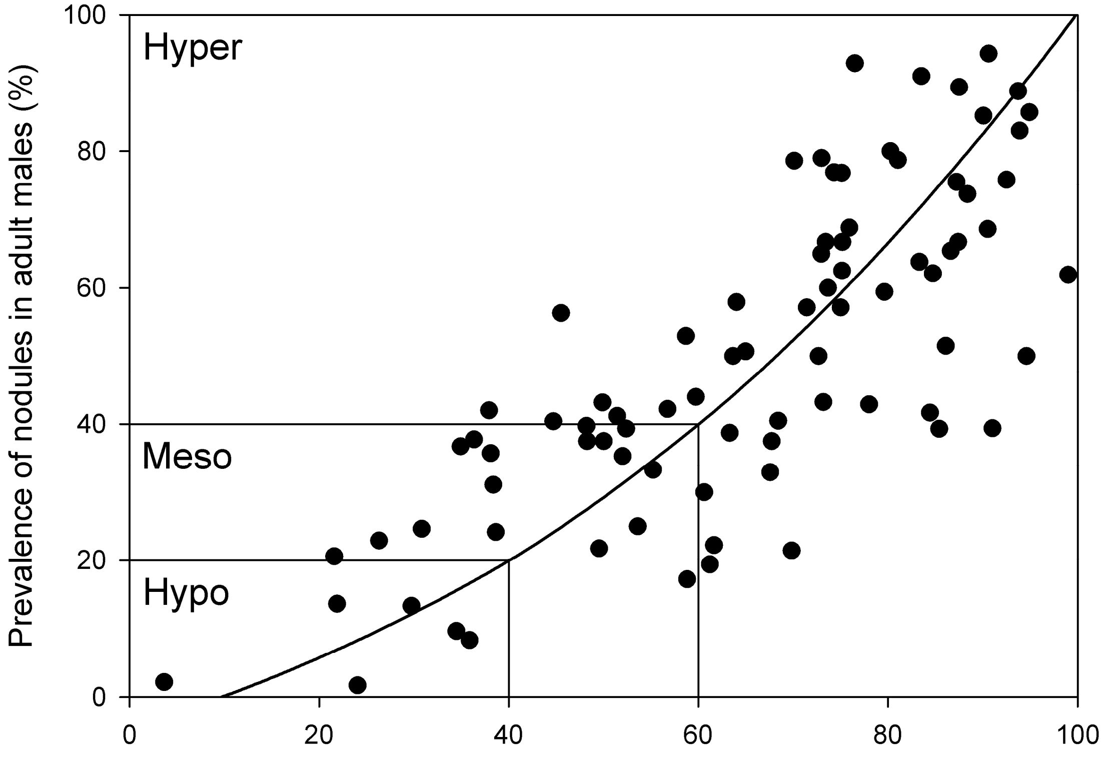

S4 Modeling trends in infection and morbidity in ONCHOSIM

Trends in prevalence of infection, blindness, visual impairment and mortality were simulated with the ONCHOSIM model [5,6]. This model can simulate transmission of *O. volvulus* and development of morbidity in a community, while accounting for the effect of interventions such as mass treatment with ivermectin or vector control. For hypoendemic areas, ONCHOSIM predicts that transmission of infection is unsustainable without migration of infected flies and/or humans. Because information on migration was lacking, no simulations were performed for hypoendemic areas. Instead, we assumed that the prevalence of infection

and morbidity in hypoendemic areas was 1/3 of that in mesoendemic areas, both pre-control and during control. For non-endemic areas, we assumed that prevalence of infection and morbidity was always zero.

Table S1: Transmission parameters used for the simulation of mesoendemic and hyperendemic areas of APOC.

		Mesoendemic areas	Hyperendemic areas
Exposure heterogeneity*		3.865	4.283
Monthly biting rate**			
	January	828	1,095
	February	729	964
	March	465	615
	April	595	787
	May	601	795
	June	524	693
	July	815	1,078
	August	1,057	1,398
	September	933	1,234
	October	1,020	1,349
	November	1,163	1,538
	December	834	1,103

* Scale parameter for a gamma distribution with mean 1, which models individual heterogeneity in exposure to infection. In other words, the average (expected) number of fly bites for a person of a certain sex and age is multiplied with an individually fixed index, which has been drawn from the mentioned gamma distribution.
** Average number of fly bites per person per month. These figures are proportional to biting rates observed in Asubende, Ghana, assuming that these seasonal patterns in biting rates are representative for other sites as well.

S5 Calibration of ONCHOSIM parameters for transmission

ONCHOSIM was calibrated to reproduce the pre-control levels of infection in each endemicity category, as estimated from the REMO data (S3.2). However, the REMO data are based on nodule prevalence in adult males, whereas ONCHOSIM provides output on prevalence and load of microfilariae in the skin. Therefore, we translated the estimated mean pre-control nodule prevalence to mf prevalence (standardized to 5+ population of the OCP reference population), based on a simple association derived from previously published data (Figure S2) [4]. The association between nodule prevalence and mf prevalence was characterized for mesoendemic and hyperendemic areas as follows:

$$\text{for nodule prevalence} \geq 20\% \text{ and} < 40\%,\ p_{mf} = p_{nod} + 20\,;$$

$$\text{for nodule prevalence} \geq 40\%,\ p_{mf} = \frac{3}{4}\left(p_{nod} + 40\right);$$

where p_{mf} and p_{nod} represent standardized mf prevalence and nodule prevalence in adult males, respectively.

Based on an analysis of the REMO data, combined with the translation of nodule prevalence to mf prevalence described above, we assumed that the mean pre-control, standardized mf prevalences in mesoendemic and hyperendemic areas were 49% and 76% respectively (corresponding to nodule prevalences of 29% and 61%, respectively). These mf prevalences were simulated in ONCHOSIM, using parameter values in Table S1. A description of the technical implementation of these parameters can be reviewed in an earlier publication [5].

S6 Calibration of ONCHOSIM parameters for eye disease

Following the WHO criteria for blindness and visual impairment, we defined blindness as visual acuity of less than 3/60 or a restriction of visual field to less than 10° in the better eye. According to the same criteria, we defined visual impairment as visual acuity of less than 6/18 but better than 3/60 in the better eye. We assumed that blindness and visual impairment are irreversible conditions, which is supported by a Cochrane review of placebo-controlled trials that found no statistically significant effect of ivermectin on functional vision loss [7], even though some early eye lesions may respond to ivermectin treatment [8]. We assumed that blindness reduces the remaining life expectancy by 50%, based on trends in blindness in OCP in West Africa (S6) [9]. For visual impairment we assumed no reduction in life expectancy.

ONCHOSIM predicts the development of eye disease as a function of cumulative exposure to infection, reflecting an accumulation of damage in the eye. If a simulated individual's cumulative mf-count passes a critical threshold level, he or she turns visually impaired or blind. In ONCHOSIM, the actual threshold is assumed to vary randomly between individuals, reflecting variation in individual susceptibility. Further, ONCHOSIM models excess mortality due to blindness by reducing the remaining life expectancy of people who have turned blind by a mean factor, again allowing some individual variation.

In ONCHOSIM, visual impairment and blindness could not be modeled simultaneously (i.e., it was possible to define one threshold for cumulative exposure to infection at a time). Therefore, we first modeled blindness; next, we modeled all visual impairment (including blindness) by lowering the value of the threshold. The excess mortality (specified as reduction in remaining life-expectancy) was adjusted accordingly. The prevalence of visual impairment, excluding blindness, was estimated by subtracting the predicted prevalence of blindness from the predicted prevalence of all visual impairment (including blindness). Because visual impairment and blindness were modeled in separate simulations, the simulated populations with blindness and visual impairment were not exactly comparable. However, differences were not large, and were deemed acceptable.

It is commonly accepted that the severity of eye disease is different for the forest and savanna types of onchocerciasis. Therefore, the thresholds for blindness and visual impairment were determined separately for savanna and forest areas. Excess mortality among blind people was assumed to be equal in forest and savanna areas.

S6.1 Disease threshold and excess mortality for savanna type of onchocerciasis

For onchocercal blindness in savanna areas, we calibrated ONCHOSIM using published data from OCP [10]. To fit a threshold value for blindness to these data, we varied the threshold and model parameters for transmission (relative biting rate and exposure heterogeneity) over a wide range of values and compared the observed (OCP data) and model-predicted association between prevalence of mf and blindness (or visual impairment) in a standardized population of 5 years and older. We assumed that the variability in exposure increased when the average monthly biting rate was lower. (This association was based on earlier unpublished work done by Anton Plaisier; when he compared data from Folonzo, Tiercoura and Asubende (Ghana); relatively low-endemic situations could only be simulated with a combination of low relative biting rates and high exposure heterogeneity.) A good fit for savanna blindness was obtained with a disease threshold for the cumulative mf count of 4000 (Figure S3).

Because in contrast to blindness, there was little literature data available about the association between visual impairment and mf prevalence in savanna areas, we based the threshold for visual impairment on a documented ratio of visual impairment and blindness in savanna areas. In pre-control, hyperendemic savanna areas of OCP, the pre-control prevalence of visual impairment has been reported to be 1.8 times the prevalence of blindness [4]. This pattern was reproduced in ONCHOSIM with a threshold value of 2800. With this value, the

predicted prevalence of eye disease (i.e. visual impairment) was 1.8 times the predicted prevalence of blindness, at mf prevalence of 73% (which was the mean prevalence of infection in hyperendemic OCP areas).

Figure S3: Goodness-of-fit of ONCHOSIM to OCP data on the association between infection and blindness and visual impairment in savanna areas. Blindness data were obtained from Remme et al (1989). For visual impairment, ONCHOSIM was calibrated so that the prevalence of visual impairment was 1.8 times the prevalence of blindness in hyperendemic areas, represented by mf prevalence 73% [4].

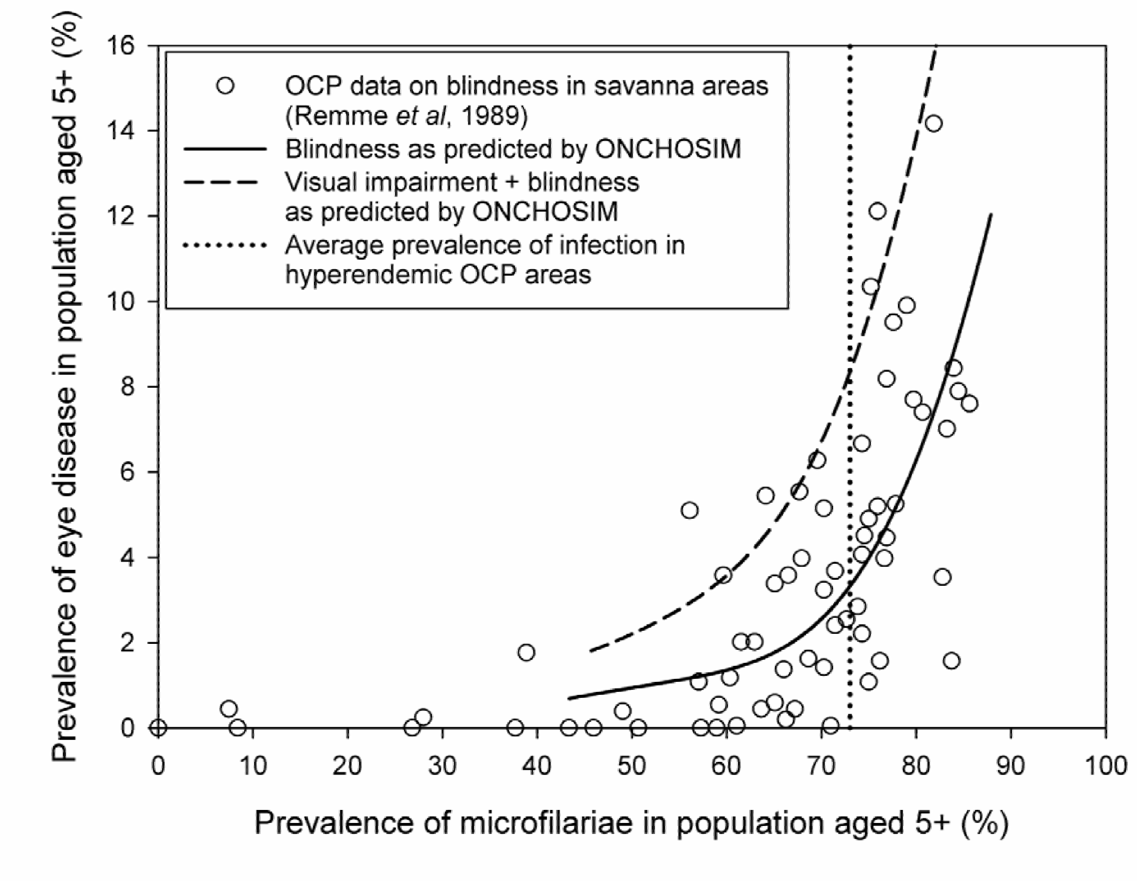

The parameter value for excess mortality (reduction in remaining life expectancy) was based on OCP data on trends in blindness during vector control. Part of these data (first 7-8 years) have been previously published [9]; additional follow-up data were kindly provided by Dr Y. Dadzie. The data pertain to ten villages in Burkina Faso, Côte d'Ivoire and Ghana for which longitudinal data was available and history of vector control was known. In these villages, the pre-control mf prevalence was between 70% and 90%. Assuming that vector control reduced the biting rate to zero (an assumption previously used to successfully predict the impact of the OCP [6]), we used ONCHOSIM to predict the trend in prevalence of blindness (Figure S4). This was done for several values of the relative biting rate and exposure heterogeneity that predict mf prevalences between 70% and 90% (top and bottom dashed lines in Figure S4). The mean trend of blindness in these simulations was compared to the data for a range of parameter values for excess mortality due to blindness. A mean 50% reduction in remaining life expectancy in blind people could adequately predict the observed trend in the OCP data. This value was allowed to vary between individuals (uniform distribution, range 0 – 100%)

In simulations for visual impairment including blindness, the parameter for excess mortality was set to 20% (uniform distribution, range 0 – 40%). Assuming that there is no

excess mortality in people with low vision, and taking account of the fact that the number of people with low vision is 1.8 times the number of blind people, 20% is roughly equal to 50% times 1.0 / (1.0+1.8).

Figure S4: Trend in prevalence of blindness in the original OCP area and the trend as predicted by ONCHOSIM, assuming that the remaining life expectancy is halved at onset of blindness. See text for further explanation. Data for the first 7-8 years have been previously published [9]; additional follow-up data were kindly provided by Dr K.Y. Dadzie.

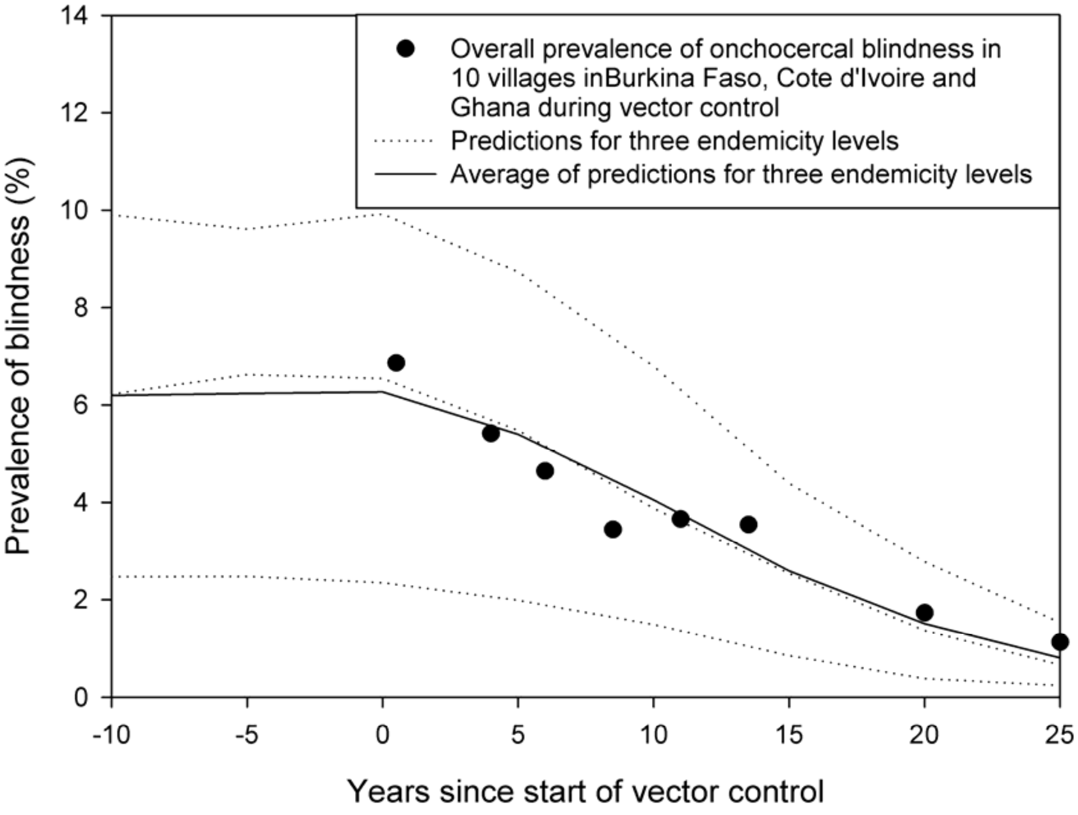

S6.2 Disease threshold for forest/mixed type of onchocerciasis

There is less information about the association between infection and blindness for the forest type of onchocerciasis than for the savannah type. Considering that the prevalence of blindness and visual impairment is much lower in forest/mixed areas than in savannah areas, for the entire range of mf prevalences, we need higher disease thresholds. We took the following approach in calibrating the disease threshold for the forest/mixed type of onchocerciasis.

We collated available literature data on onchocercal blindness and infection in non-savanna areas (forest and mixed forest-savanna areas) [4,11-16]. Because the literature data varied with respect to the methods to measure blindness, the age groups in which blindness prevalence was measured and the indicator of infection (microfilariae in the skin or palpable nodules), we standardized the data before calibrating ONCHOSIM. In many studies only the central vision was tested whereas onchocerciasis also affects the peripheral vision. According to the WHO criteria, persons can also be functionally blind if the peripheral vision is affected. When this was not taken into account, we multiplied the reported prevalence of blindness by 4/3, assuming that 25% of functional blindness had been missed, as previously estimated [4]. If reported blindness was due to any cause, a background prevalence of non-onchocercal

blindness of 0.96% was assumed, as previously estimated [16]. If nodule prevalence in adult males was reported, we translated it to mf prevalence in the general population, using the association described in section S5. Next, we fitted the threshold levels for blindness and low vision in ONCHOSIM to the standardized data. The parameters for excess mortality among the blind and the association between biting rate and exposure heterogeneity were assumed equal to the model calibration for the savanna areas. A good fit was obtained with a threshold value of 10000 for blindness and of 5500 for visual impairment. The goodness-of-fit is shown in Figure S5.

Figure S5: Goodness-of-fit of ONCHOSIM to data on the association between infection and blindness and visual impairment in forest and mixed forest / savanna areas.

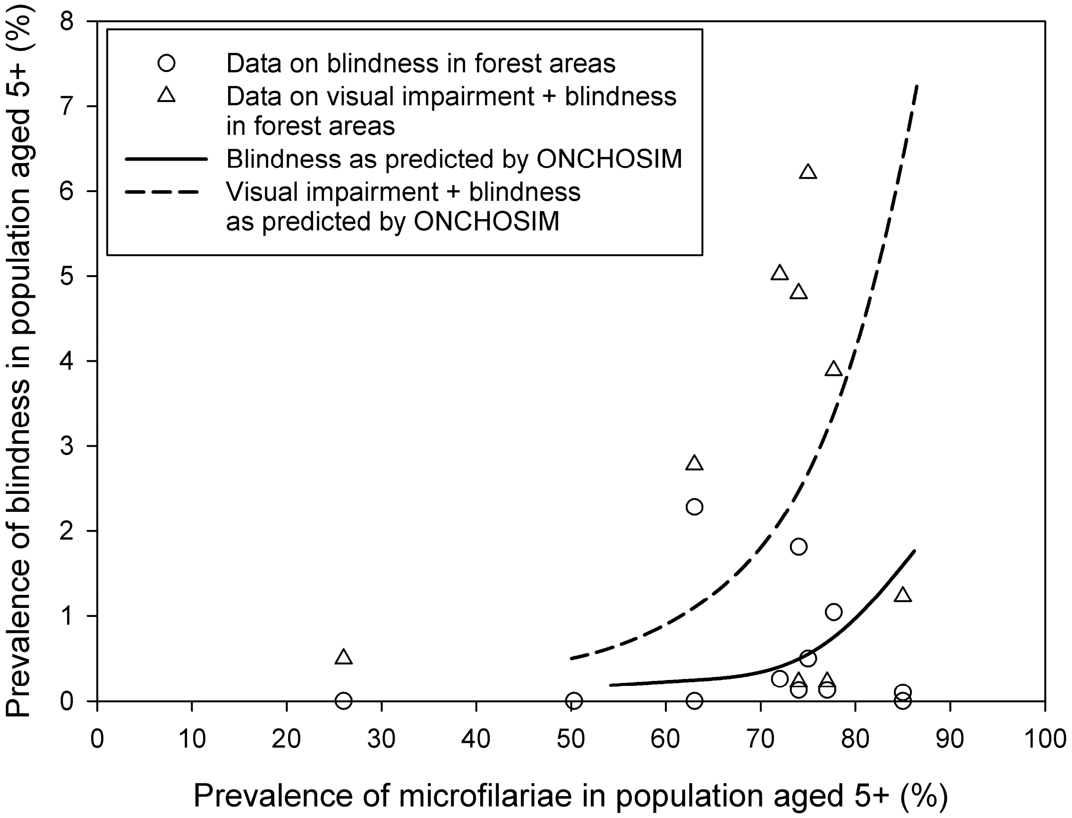

S7 Calibration of model parameters for troublesome itch

We estimated the prevalence of itch from the prevalence of infection, as predicted by ONCHOSIM. For the pre-control situation, we could have simply related the prevalence of itch to the prevalence of microfilariae, assuming that itch is a direct effect of the presence of microfilariae in the skin; empirical data are available about this relationship. However, from ivermectin trials we learn that this direct link between presence of mf and itch does not hold during ivermectin treatment; prevalence and skin load of microfilariae in the population drop sharply almost instantly after treatment, whereas the reduction in prevalence of itch is smaller and moreover, delayed compared to the drop in mf load and prevalence [17]. This means that after ivermectin treatment, some people can still experience itch, even though there microfilarial loads have dropped drastically. Furthermore, this means that linking itch to microfilaria as predicted by ONCHOSIM is probably not the best way to predict trends in itch. Because adult worms have a longer life span than microfilariae and because ivermectin

treatment does not (or only marginally) affect adult worm viability, we linked itch to the presence of adult worms in the body. Because at this point we have not yet accounted for the effect of ivermectin on itch, we refer to this predicted prevalence of itch as 'potential' prevalence of itch, referring to the prevalence of itch in the fraction of the population which has not participated in mass treatment in a given year. For the fraction of the population that was actually treated with ivermectin, we corrected the potential prevalence of itch for the effect of ivermectin, based on literature data.

The association between prevalence of adult female worms and itch was analyzed in the following steps. First, we determined the statistical association between nodule prevalence in adult males and potential prevalence of itch in the general population, based on data from a multi-country study [18]. Second, we determined the statistical association between prevalence of adult female worms and potential prevalence of itch in the general population by substituting nodule prevalence for standardized mf prevalence (based on the association between the two as described in S5), and next substituting standardized mf prevalence for prevalence of adult female worms (based on the association between the two as predicted by ONCHOSIM). Last, we determined the average year-round reduction in prevalence of itch in treated individuals, based on literature [17].

S7.1 Statistical association between nodule prevalence and itch

Using data from a multi-country study on prevalence of infection and skin-disease, we estimated the association between nodule prevalence and the prevalence of troublesome itch for the forest-type of onchocerciasis [18]. As there is no evidence for a difference in patterns of itch between savanna and forest areas, and for lack of data, we assumed that this association also holds in savanna areas.

We re-analyzed the original raw data to obtain village-specific estimates for the nodule prevalence in adult males and the prevalence of itch in the whole population. Estimates of the nodule prevalence in adult males could be readily obtained from the data. However, the crude estimates of troublesome itch in the whole population were biased because younger age groups were underrepresented and elderly were overrepresented in the data. Therefore, we standardized the data to the United Nations World Population Prospects standard population for Sub Sahara Africa (2003 Revision). The standardized prevalence levels were always lower than the crude ones.

Next, the age-standardized data were further analyzed to estimate the background prevalence of itch at zero prevalence of infection (i.e. prevalence of itch that is not caused by onchocerciasis). A regression line was fitted to the data by means of orthogonal linear regression (Figure S6). This regression method corrects (to some extent) for non-systematic misclassification of exposure (nodule prevalence), which leads to dilution-bias in case of ordinary linear regression. Ignoring this non-systematic misclassification would lead to an underestimation of the strength of the relationship between onchocerciasis infection and itch and an inflated estimate of the background non-onchocercal itch. In orthogonal regression, the correction for dilution-bias is based on a (assumed) ratio (lambda) of the measurement error (variance) in the exposure and outcome variables. We assumed that the variance of the measurement errors in nodule prevalence and prevalence of troublesome itch were equal (lambda = 1). The slope of the regression line was estimated at 0.416 (increase in potential prevalence of itch for every 1% increase in nodule prevalence); the intercept was estimated at 5.888 (background prevalence of itch).

The background prevalence of itch was therefore estimated at 5.9%. However, we also wanted to take into account that some of the people with itch from other causes may in addition suffer from onchocercal itch. Therefore, we did not simply subtract the 5.9% background prevalence from the estimated prevalence of all-cause itch. Instead, we assumed

that in addition to the predicted prevalence of potential onchocercal itch, a certain proportion of people suffering from itch due to other causes suffered (partly) from onchocercal itch. This proportion was assumed to be equal to the prevalence of onchocercal itch in the population without itch from other causes, leading to the following equation for total predicted prevalence of potential itch: *slope**nodule prevalence *100/(100 – *intercept*).

Figure S6: The association between nodule prevalence in adult males (x-axis) and the prevalence of troublesome itch (y-axis), based on standardized data (bullets) from previously published work by Murdoch *et al* [18]. The regression line representing the association was estimated by means of orthogonal regression, assuming that the variance of the measurement errors in nodule prevalence and prevalence of troublesome itch were equal (lambda = 1).

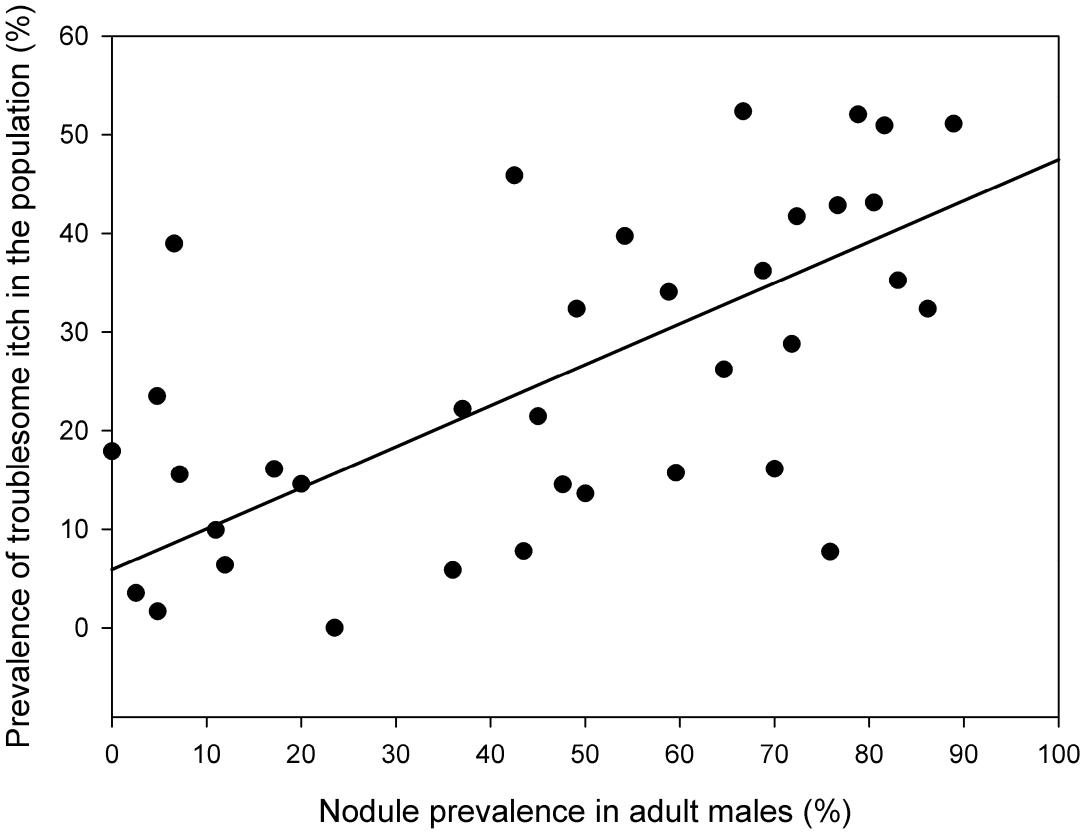

S7.2 The association between prevalence of itch and adult female worms

As mentioned earlier, we linked the association between nodule prevalence in adult males and itch in the general population to ONCHOSIM predictions for standardized mf prevalence in the population aged five and above, using the known association between nodule prevalence and mf prevalence (S5). By calculating the prevalence of itch for a range of simulated mf prevalences, and plotting these prevalences of itch against the concomitantly simulated prevalences of adult female worms, we could determine a statistical association between prevalence of itch and adult female worms in the general population (Figure S7).

Note that the simulated itch prevalence was almost linearly related to the prevalence of adult worms. However, as ONCHOSIM predicts that independently sustained transmission of infection in hypoendemic areas is impossible, we could not calculate prevalences of itch for situations that corresponded with low prevalence of adult female worms. Instead, we assumed that the regression line for this association passes through the origin, using the following equation:

$$p_{itch} = a \times p_{worms} + b \times \left(1 - \exp\left((p_{worms}/100)^2\right)\right),$$

where p_{itch} and p_{worms} are prevalence of itch and adult female worms (0 – 100% scale), respectively. The values of parameters a and b were estimated at -0.043 and -45.532, respectively. For prevalence of adult female worms close to zero, this equation can take on negative values; in that case, we assumed that the itch prevalence was zero.

Using this final equation, we predicted the 'potential' prevalence of itch in the general population, based on its current status of infection (prevalence of adult female worms) and as if it had not (yet) been treated with ivermectin during that year.

Figure S7: The modeled relationship between prevalence of adult female worms and onchocerciasis-related troublesome itch in the population. Bullets represent ONCHOSIM predictions for prevalence of adult female worms and the associated itch, calculated from the concomitantly predicted standardized prevalence of microfilariae in the skin. The association between prevalence of adult female worms and itch (solid line) was determined by means of non-linear regression. In the sensitivity analysis, parameter *b* was allowed to vary by ±25%, resulting in a stronger or weaker association between prevalence of adult worms and prevalence of onchocerciasis-related troublesome itch (dashed lines).

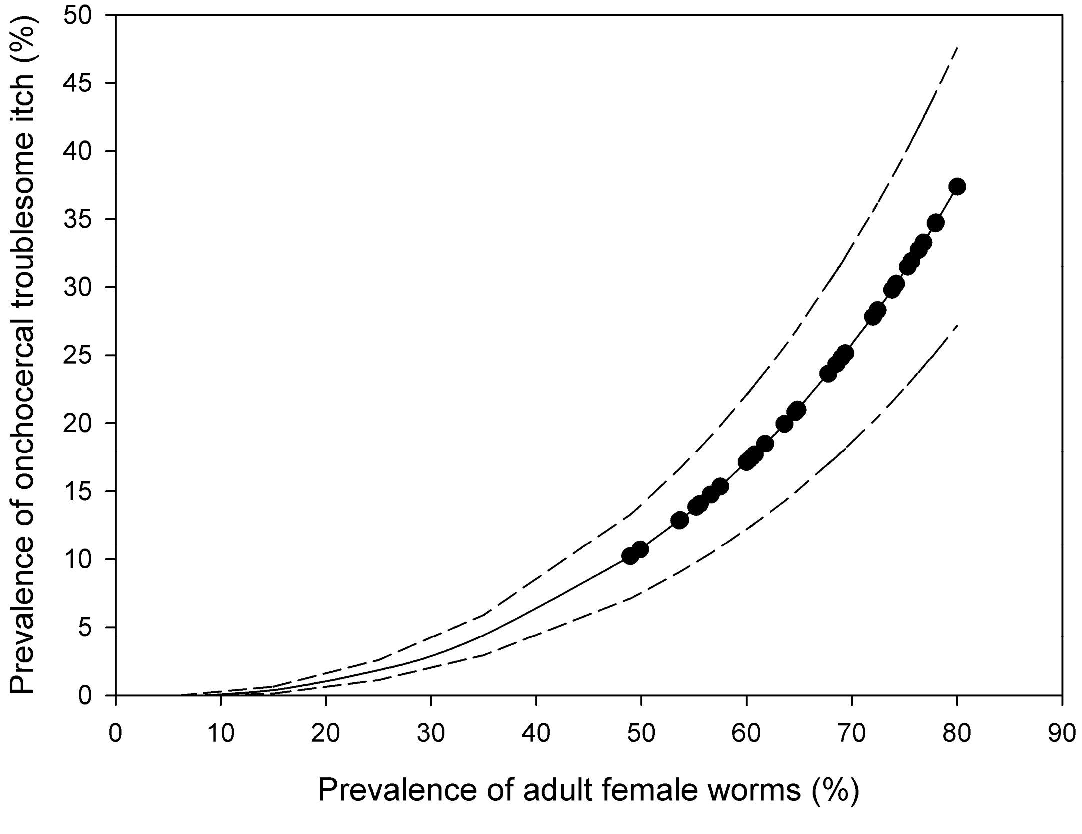

S7.3 The effect of ivermectin on prevalence of itch

Based on literature [17], we assumed that ivermectin reduces the average year-round prevalence of itch by 30% in treated individuals. This figure is based on the observed pattern in the relative reduction of prevalence of itch at 3, 6, 9, and 12 months after a single treatment of ivermectin. When plotting the relative reduction of prevalence of itch over time (relative reduction of 5.6%, 44.9%, 46.3%, and 31%, respectively) and connecting these data points

with straight lines, the area under the curve was approximately 30%, representing the average year-round reduction in prevalence of itch due to ivermectin.

The estimated prevalence of itch among treated individuals was calculated by first calculating the ‘potential’ prevalence of itch, based on predicted prevalence of adult female worms just before mass treatment (which was assumed to take place at the start of the year), and multiplying it with 0.7 (30% reduction). The average year-round prevalence of itch among non-treated individuals was estimated from the mid-year prevalence of adult female worms, without correction for an effect of ivermectin. Finally, the average year-round prevalences of itch in the treated and untreated fractions of the population were averaged, weighted for the size of each population fraction.

S8 Calculation of the burden of disease

For each year, we calculated the number of disability adjusted life years (DALYs) lost due to onchocerciasis in the APOC area, as the sum of years of life lived in disability (YLD) and year of life lost (YLL) due to excess mortality from blindness. YLD were calculated by multiplying number of prevalent cases with previously published disability weights; i.e., 0.594 for blindness, 0.282 for visual impairment and 0.068 for troublesome itch [19]. YLL apply to blindness only and were calculated based on incident cases of blindness (i.e. lost future life years). The annual number of incident cases of blindness was calculated as the difference between the number of blind cases in year *t* and the number of blind cases in year (*t*-1) that were expected to have survived up to year *t*, based on the average remaining life-expectancy at onset of blindness. The latter was estimated by determining the average age of onset of blindness in ONCHOSIM (in a situation without mass treatment), and calculating the associated average remaining life-expectancy for a healthy person (which was 16 years), and combining this with the 50% reduction in life-expectancy due to blindness. Consequently, every incident case of blindness was assumed to have an average remaining life expectancy of 8 years, meaning that 7 out of 8 prevalent cases of blindness were assumed to survive each year. Also following from this, every incident case of blindness was attributed 8 YLL in terms of burden of disease.

S9 Sensitivity analysis

To investigate the impact of model assumptions on the estimated health impact, we performed univariate and multivariate sensitivity analyses. We included model assumptions that were expected to possibly have an important impact on the estimated health impact. In the univariate sensitivity analysis, we repeated the original analysis, but with (plausible) extreme values for each of the following model and data-derived parameters (extreme values between brackets): size of population at risk (±10%), pre-control levels of infection (±10%), therapeutic coverage (±10%), the association between exposure to infection and development of eye disease (±25% in the required cumulative exposure to infection for development of eye disease, Figure S8), the association between prevalence of adult female worms and troublesome itch (parameter *b* ±25%, Figure S7), effect of ivermectin on adult female worms (26% or 40% permanent reduction in fecundity, instead of 35%, based on a previously published 95%-confidence interval for this parameter value [20]), effect of ivermectin on itch (reduction in prevalence of 20% or 40%, instead of 30%), the years of life lost per incident case of blindness (6 or 10 years, instead of 8), and levels of infection and morbidity in hypoendemic areas as fraction of mesoendemic areas (1/10 or 1/2, instead of 1/3).

In the multivariate sensitivity analysis, we repeated the original analysis 200 times, while letting all selected parameters vary in each analysis. This analysis allowed for possible interaction between parameters; e.g., lower mass treatment coverage combined with higher pre-control infection levels might result in a drastically lower estimated health impact.

Parameter values were varied by randomly drawing values from triangular distributions, which were defined by a mode equal to the parameter value used in the main analysis, and minimum and maximum values equal to the extreme values used in the univariate sensitivity analysis. Assuming that these triangular distributions are an adequate reflection of the uncertainty in the parameter values, we made a crude estimate of the uncertainty in the estimated health impact of APOC by taking the 2.5 and 97.5 percentiles of the results from the 200 repeated analyses.

S10 Acknowledgements

We thank Drs. M.C. Asuzu, M. Hagan, W.H. Makunde, P. Ngoumou (deceased), K.F. Ogbuagu, D. Okello, G. Ozoh and J.H.F. Remme for their contributions to pre-control data on nodule prevalence and prevalence of itch. We thank Dr. Y. Dadzie for contributing data on trends in blindness during vector control. We thank Dr. A. Hopkins, director of the Mectizan Donation Program, for providing estimates of cost per tablet ivermectin and average number of tablets needed per ivermectin treatment.

Figure S8: Association between prevalence of infection and eye disease as predicted by ONCHOSIM in the main analysis (middle line in each panel) and the univariate sensitivity analysis (outer lines in each plot), compared to the data (open circles). For the univariate sensitivity analysis it was assumed that the required cumulative exposure to infection for the development of eye disease was 25% lower or higher than assumed in the main analysis.

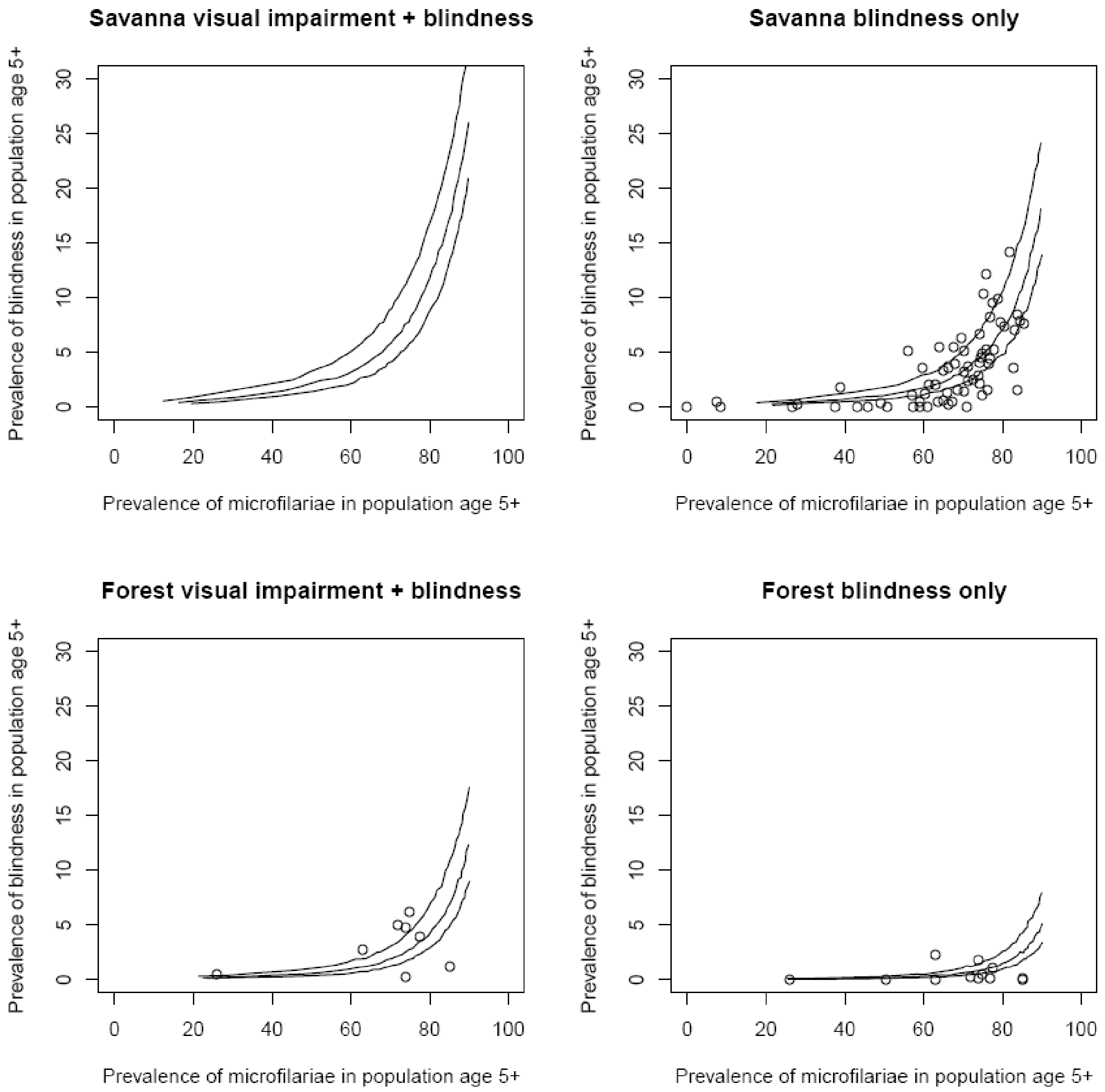

References

1. Ngoumou P, Walsh JF (1993) A Manual for Rapid Epidemiological Mapping of Onchocerciasis. Geneva: UNDP/World Bank/WHO.

2. Noma M, Nwoke BE, Nutall I, Tambala PA, Enyong P, et al. (2002) Rapid epidemiological mapping of onchocerciasis (REMO): its application by the African Programme for Onchocerciasis Control (APOC). Ann Trop Med Parasitol 96 Suppl 1: S29-39.

3. Yee TW, Wild CJ (1996) Vector generalized additive models. Journal of the Royal Statistical Society Series B-Methodological 58: 481-493.

4. Remme JHF (2004) The Global Burden of Onchocerciasis in 1990. Geneva: WHO.

5. Plaisier AP, van Oortmarssen GJ, Habbema JD, Remme J, Alley ES (1990) ONCHOSIM: a model and computer simulation program for the transmission and control of onchocerciasis. Comput Methods Programs Biomed 31: 43-56.

6. Plaisier AP, Alley ES, van Oortmarssen GJ, Boatin BA, Habbema JD (1997) Required duration of combined annual ivermectin treatment and vector control in the Onchocerciasis Control Programme in west Africa. Bull World Health Organ 75: 237-245.

7. Ejere H, Schwartz E, Wormald R (2001) Ivermectin for onchocercal eye disease (river blindness). Cochrane Database Syst Rev: CD002219.

8. Abiose A (1998) Onchocercal eye disease and the impact of Mectizan treatment. Ann Trop Med Parasitol 92 Suppl 1: S11-22.

9. Dadzie KY, Remme J, Rolland A, Thylefors B (1986) The effect of 7-8 years of vector control on the evolution of ocular onchocerciasis in West African savanna. Trop Med Parasitol 37: 263-270.

10. Remme J, Dadzie KY, Rolland A, Thylefors B (1989) Ocular onchocerciasis and intensity of infection in the community. I. West African savanna. Trop Med Parasitol 40: 340-347.

11. Brown R, Shannon R (1989) Prevalence, intensity and ocular manifestations of Onchocerca volvulus infection in Dimbelenge, Zaire. Ann Soc Belg Med Trop 69: 137-142.

12. Henry MC, Maertens K (1990) The onchocerciasis focus at Kinsuka/Kinshasa (Republic of Zaire) in 1985. II. Parasitological and clinical aspects. Ann Trop Med Parasitol 84: 493-502.

13. Whitworth JA, Gilbert CE, Mabey DM, Maude GH, Morgan D, et al. (1991) Effects of repeated doses of ivermectin on ocular onchocerciasis: community-based trial in Sierra Leone. Lancet 338: 1100-1103.

14. Whitworth JA, Gilbert CE, Mabey DM, Morgan D, Foster A (1993) Visual loss in an onchocerciasis endemic community in Sierra Leone. Br J Ophthalmol 77: 30-32.

15. Kayembe DL, Kasonga DL, Kayembe PK, Mwanza JC, Boussinesq M (2003) Profile of eye lesions and vision loss: a cross-sectional study in Lusambo, a forest-savanna area hyperendemic for onchocerciasis in the Democratic Republic of Congo. Trop Med Int Health 8: 83-89.

16. Resnikoff S, Pascolini D, Etya'ale D, Kocur I, Pararajasegaram R, et al. (2004) Global data on visual impairment in the year 2002. Bull World Health Organ 82: 844-851.

17. Brieger WR, Awedoba AK, Eneanya CI, Hagan M, Ogbuagu KF, et al. (1998) The effects of ivermectin on onchocercal skin disease and severe itching: results of a multicentre trial. Trop Med Int Health 3: 951-961.

18. Murdoch ME, Asuzu MC, Hagan M, Makunde WH, Ngoumou P, et al. (2002) Onchocerciasis: the clinical and epidemiological burden of skin disease in Africa. Ann Trop Med Parasitol 96: 283-296.

19. World Health Organization (2004) Global Burden of Disease update 2004: disability weights for diseases and conditions. Geneva: World Health Organization.

20. Plaisier AP, Alley ES, Boatin BA, Van Oortmarssen GJ, Remme H, et al. (1995) Irreversible effects of ivermectin on adult parasites in onchocerciasis patients in the Onchocerciasis Control Programme in West Africa. J Infect Dis 172: 204-210.
